# Stressed portfolio optimization with semiparametric method

**DOI:** 10.1186/s40854-022-00333-w

**Published:** 2022-03-14

**Authors:** Chuan-Hsiang Han, Kun Wang

**Affiliations:** 1 Department of Quantitative Finance, National Tsing Hua University, Hsinchu, 30013 Taiwan; 2 International Intercollegiate Ph.D. Program, National Tsing Hua University, Hsinchu, 30013 Taiwan

**Keywords:** Portfolio optimization, Tail risk, Semiparametric method, Kernel method, Copula method, Risk measure, Risk-sensitive value measure, Scaling effect

## Abstract

Tail risk is a classic topic in stressed portfolio optimization to treat unprecedented risks, while the traditional mean–variance approach may fail to perform well. This study proposes an innovative semiparametric method consisting of two modeling components: the nonparametric estimation and copula method for each marginal distribution of the portfolio and their joint distribution, respectively. We then focus on the optimal weights of the stressed portfolio and its optimal scale beyond the Gaussian restriction. Empirical studies include statistical estimation for the semiparametric method, risk measure minimization for optimal weights, and value measure maximization for the optimal scale to enlarge the investment. From the outputs of short-term and long-term data analysis, optimal stressed portfolios demonstrate the advantages of model flexibility to account for tail risk over the traditional mean–variance method.

## Introduction

Several historical episodes, such as the financial crisis and COVID-19, have posed new challenges for investment management in unknown and unprecedented tail risks. A large body of literature on econometric research exploits the validation of various financial models and risk measures, such as value-at-risk (VaR) and conditional value at risk (CVaR) for risk management (Jorion 2007). We extend the use of these risk measures (Artzner et al. 1999) for portfolio optimization using a novel semiparametric modeling method under stressed scenarios. The scaling effect of stressed portfolios is also a concern. Risk-sensitive value measures (Miyahara 2010) were adopted to maximize the optimal scale for a given portfolio strategy.

The proposed semiparametric modeling method is constructive and consists of two estimation procedures: the nonparametric kernel method for marginal distributions and a parametric copula method for their joint distribution. This semiparametric method builds up a more complex dependence between portfolio constituents than traditional Gaussian models that can be used to exploit tail risks.

From both experimental and theoretical perspectives, we find that the proposed optimal stressed portfolio and the semiparametric method perform better than Markowitz’s mean–variance method (Markowitz 1952). From an experimental perspective, our implementation of the stressed portfolio optimization relies on a rolling window approach and checks its robustness. In addition, from a theoretical perspective, the risk-sensitive value measure (RSVM) is equipped with more properties for general heavy-tail distribution than Markowitz’s mean–variance model, thus making mean–variance a special case in the risk-sensitive value measure.

The remainder of this paper is organized as follows: “Literature review” section provides a literature review, particularly on the nonparametric kernel method and the parametric copula method. “The semiparametric method” section generates non-Gaussian distributed portfolios using the proposed semiparametric method with two parts. First, we construct the marginal distribution of each constituent asset by nonparametric estimation with cross-validation to obtain the optimal bandwidth of a kernel function and its perturbation analysis. The alternative part estimates the parameters of copula functions by full maximum likelihood estimation (MLE). “Stressed portfolio optimization and its scaling effect” section solves the optimal weights of the portfolio using the semiparametric method by minimizing risk measures, such as VaR and CVaR. The scaling effect is then optimized by maximizing the risk-sensitive value measures. “Empirical studies and data analysis” section presents the data set, intensive empirical results, and a comparison between the stressed portfolio and the traditional mean–variance method. We conclude the paper in “Conclusion” section.

## Literature review

There are two major directions for tail risk estimation: modeling the return distribution and capturing the volatility process. For the former direction, various techniques are employed for modeling the entire return distribution or just the tail areas, including known parametric distribution, kernel density approximation, and extreme value theory (Tsay 2010). The latter direction mostly relies on discrete-time volatility models, such as the exponentially weighted moving average model (EWMA) and autoregressive general conditional heteroskedasticity (GARCH) model to capture the volatility process. See Jondeau et al. (2007) for further details.

Traditional modeling methods in financial management often rely on the Gaussian distribution by virtue of closed-form solutions for mean–variance analysis (Fu et al. 2021), the optimal risk measure, and so on. There are other risk measures such as the Entropic Value-at-Risk (Mills et al. 2017). However, some stylized facts of heavy tails and asymmetry among empirical distributions expose extra risk for the fraud of initial assumptions. In contrast, we relax the Gaussian assumption using a semiparametric method, which renders flexible distributions to describe more details and properties for unknown tail risks.

Distinct from previous studies on financial modeling, the aim of this study is to build up the joint distribution of portfolios in high dimensions without assumptions of each underlying asset distribution. This innovative construction of a joint distribution is based on nonparametric estimation (Robinson 1983) and the copula method (Cherubini et al. 2004, 2011). Nonparametric estimation with a kernel function is adopted to estimate the probability density function of each underlying asset, and the parametric copula method is used to describe a joint distribution between the assets of the portfolio. Among nonparametric estimations, several studies exploit the optimal kernel functions and bandwidth in the estimation by Robinson (1983). There is no universally accepted approach to select the optimal kernel function that has little influence on the estimation results. We concentrate on the selection of the optimal bandwidth using cross-validation theory (Horová et al. 2012). A bias estimation for the perturbed optimal bandwidth is derived. Regarding the parametric copula method, there are two primitive families of copula functions: elliptical and Archimedean copula (Nelsen 1999). The multivariate copula method builds up the dependence on portfolio constituents.

Notably, the proposed semiparametric modeling method is static, in comparison to dynamical multivariate models such as the GARCH DCC model (Engle 2002) in discrete time or stochastic volatility matrix model in continuous time (Mancino et al. 2017; Han 2018). The former static model can be quite complex in its structure, whereas the latter dynamic model advances its prediction capability. The stressed portfolio optimization problem under the static model is the focus of this study. Owing to the complexity of financial modeling, computational schemes such as optimization solvers and the Monte Carlo estimator by simulations are utilized. There are several techniques for solving portfolio optimization models (Esfahanipour and Khodaee 2021), particle swarm optimization (PSO), and so on. Motivated by Babazadeh and Esfahanipour (2019), the optimization algorithm genetic algorithm (GA) was used to solve risk measure minimization problems using MATLAB’s package. However, its counterpart dynamic version requires solving high-dimensional nonlinear HJB-type partial differential equations (Fleming and Soner 2006) in continuous time.

### Marginal distribution: nonparametric kernel method

A nonparametric estimation utilizes kernel functions to smooth out the shape of the distribution from discrete raw data into continuous data. The degree of smoothness has a limited relationship with kernel functions, whereas it depends on the bandwidth of the kernel.

#### Kernel function

These are many choices of kernel functions, such as Gaussian kernel, exponential kernel, and Cauchy kernel. However, the Gaussian kernel,

2.1 $$K\left(x\right)=\frac{1}{\sqrt{2\pi }}{\mathrm{e}}^{-\frac{{x}^{2}}{2}},$$

is commonly used in practice because it does not influence the asymptotic of the estimation as significant as the bandwidth used by Horová et al. (2012).

##### Definition 2.1

Suppose that there are $n$ observed values (or returns) denoted by vector $X$. The kernel estimator (*Rosenblatt-Parzen*) $\widehat{f}$ at point $x\in R$ is defined as:

2.2 $$\widehat{f}\left(x;\mathrm{h}\right)=\frac{1}{nh}\sum_{i=1}^{n}K\left(\frac{x-{X}_{i}}{h}\right)=\frac{1}{n}\sum_{i=1}^{n}{K}_{h}\left(x-{X}_{i}\right),$$

where ${K}_{h}\left(t\right)=\frac{1}{h}K\left(\frac{t}{h}\right), h>0.$ The positive number $h$ is a smoothing parameter called the bandwidth of the kernel function.

### Joint distribution: copula method

The copula method (Nelsen 1999, Cherubini et al. 2004) provides a useful tool for describing the dependence between variables. Two families of copula functions are often considered: elliptical and Archimedean copulas. Unlike the nonparametric kernel function, the copula method is parametric and contributes to the joint distribution of the portfolio from its multiple marginal distributions (Bouyé et al. 2000; Cambanis et al. 1981; Cherubini and Luciano 2001).

#### Definition 2.2

An *m*-dimension copula is a distribution function on ${[\mathrm{0,1}]}^{m}$ with standard uniform marginal distributions.

2.3 $$C\left({\varvec{u}}\right)= C \left({u}_{1} {,u}_{2} ,\dots ,{ u}_{m}\right),$$

where $C$ is called a copula function.

The copula function $C$ is a mapping of form $C{: [\mathrm{0,1}]}^{m}\to \left[\mathrm{0,1}\right].$ These are two major types of elliptical copula families: Gaussian and Student’s t copulas. Both are associated with a class of elliptical distributions.

#### The multivariate dispersion copula

The *m*-dimensional normal or Gaussian copula is derived from the *m*-dimensional Gaussian distribution. The Gaussian copula is generated from a set of correlated normally distributed variates ${v}_{1},{v}_{2}$…${v}_{m}$ using Cholesky’s decomposition, and then transforms these to uniform variables ${u}_{1}=\Phi \left({v}_{1}\right), {u}_{2}=\Phi ({v}_{2})$…${u}_{m}=\Phi ({v}_{m})$, where $\Phi$ is the cumulative standard normal; therefore, the pair $({u}_{1},{u}_{2}\dots {u}_{m})$ draws from the Gaussian copula.

The marginal distribution of each variable is standard normal, and the joint normal distribution can be defined as

2.4 $${C}_{R}^{Gaussian}\left({u}_{1} {,u}_{2} ,\dots ,{ u}_{m}\right)\equiv {\Phi }_{m}\left({\Phi }^{-1}\left({u}_{1}\right),\dots ,{\Phi }^{-1}\left({u}_{m}\right);R\right),$$

where $R$ is the m-dimensional covariance matrix, and ${\Phi }_{m}$ is the cumulative multivariate normal distribution function in dimension $m$.

For the multivariate Gaussian copula (MGC), let $R$ be a symmetric, positive define matrix with $\mathrm{diag}\left(R\right)={(\mathrm{1,1}\dots 1)}^{T},$ and the corresponding density function of (2.4) is,

2.5 $${c}_{R}^{Gaussian}\left(\Phi \left({x}_{1}\right),\dots ,\Phi \left({x}_{m}\right)\right)=\frac{\frac{1}{{\left(2\pi \right)}^\frac{m}{2}{\left|R\right|}^\frac{1}{2}}{exp}\left({{-\frac{1}{2}X}^{T}R}^{-1}X\right)}{\prod_{j=1}^{m}\left(\frac{1}{\sqrt{2\pi }}\mathrm{exp}\left(-\frac{1}{2}{x}_{j}^{2}\right)\right)}$$

where $R$ is the covariance matrix of vector $X,$ and $\left|\mathrm{R}\right|$ is the determinant of $\Sigma .$ Let ${u}_{j}={\Phi }\left({x}_{j}\right);$ therefore, ${x}_{j}={\Phi }^{-1}\left({u}_{j}\right).$ This copula density function can be rewritten as given below:

2.6 $${c}_{R}^{Gaussian}\left({u}_{1} {,u}_{2} ,\dots ,{ u}_{m}\right)=\frac{1}{{\left|R\right|}^\frac{1}{2}}exp\left({{-\frac{1}{2}\varsigma^{T}(R}^{-1}-I)}\varsigma\right)$$

where $$\varsigma=({\Phi }^{-1}\left({u}_{1}\right),\dots ,{\Phi }^{-1}\left({u}_{m}\right))$$.

Let ${\varvec{\mu}}={({\mu }_{1},{\mu }_{2}\dots {\mu }_{m})}^{T}$ be a positive parameter, ${\varvec{\upsigma}}={({\upsigma }_{1},{\upsigma }_{2}\dots {\upsigma }_{m})}^{T}$ be a dispersion parameter, and $\mathrm{R}$ be a correlation matrix. The multivariate dispersion copula (MDC) density is as given below:

2.7 $$f\left(X;{\varvec{\mu}},{\varvec{\sigma}},R\right)=\frac{1}{{\left|R\right|}^\frac{1}{2}}exp({{-\frac{1}{2}\varsigma}^{T}(R}^{-1}-I)\varsigma)\prod_{j=1}^{m}{{f}_{j}\left({x}_{j};{\mu }_{j},{\sigma }_{j}\right)},$$

where ${\varsigma}_{j}={\Phi }^{-1}{(F}_{j}\left({x}_{j};{\mu }_{j},{\upsigma }_{j}\right))$, and ${{f}_{j}\left({x}_{j};{\mu }_{j},{\upsigma }_{j}\right)=\frac{\partial {(F}_{j}\left({x}_{j};{\mu }_{j},{\upsigma }_{j}\right)}{\partial {x}_{j}}}$ for every set of c.d.f. ${F}_{j}\left({x}_{j};{\mu }_{j},{\upsigma }_{j}\right)$.

#### The multivariate student’s *t* copula

Similarly, the m-dimensional Student’s t-copula is derived from the m-dimensional Student’s t-distribution. Student’s t copulas are models with a heavier tail than Gaussian copulas. We denote ${\mathbf{T}}_{m}$(${\epsilon }_{1},\dots ,{\epsilon }_{m};{\varvec{R}},v$) be the joint Student’s t distribution and $\mathbf{T}(x)$ be the univariate Student’s t distributions. The Student’s t copula is defined as,

2.8 $${C}_{R,v}\left({u}_{1} {,u}_{2} ,\dots ,{ u}_{m}\right)\equiv {\mathbf{T}}_{m}\left({\mathbf{T}}^{-1}\left({u}_{1}\right),\dots ,{\mathbf{T}}^{-1}\left({u}_{m}\right);{\varvec{R}},v\right),$$

and its density function of the multivariate Student’s t copula (MTC) is,

2.9 $$\begin{aligned}{}&{\mathrm{T}}_{R,v}\left({u}_{1} {,u}_{2} ,\dots ,{u}_{m}\right)={t}_{R,v}\left({t}_{v}^{-1}\left({u}_{1}\right),{t}_{v}^{-1}\left({u}_{2}\right)\dots {t}_{v}^{-1}\left({u}_{m}\right)\right)\\ &\quad = \int\limits_{-\infty}^{t_v^{-1}({u}_{1})}\cdots\int\limits_{-\infty}^{t_v^{-1}({u}_{m})} \frac{\Gamma\left[\frac{v+m}{2}\right]|R|^\frac{1}{2}}{\Gamma\left[\frac{v}{2}\right]{v}^\frac{m}{2}\pi^\frac{m}{2}} \left(1+\frac{1}{v}{X}^{T}{\mathrm{R}}^{-1}X\right)^{-\frac{v+m}{2}}d{x}_{1}\dots d{x}_{m}\end{aligned}$$

where ${t}_{v}^{-1}$ is the inverse of the univariate cumulative distribution function of Student’s t with $v$ degrees of freedom. Using the standard representation, the copula density for multivariate Student’s t copula (Cherubini et al. 2004) is:

2.10 $${c}_{R,v}\left({u}_{1} {,u}_{2} ,\dots ,{ u}_{m}\right)={\left|R\right|}^\frac{1}{2}\frac{\Gamma \left[\frac{v+m}{2}\right]}{\Gamma \left[\frac{v}{2}\right]}{\left(\frac{\Gamma \left[\frac{v}{2}\right]}{\Gamma \left[\frac{v+1}{2}\right]}\right)}^{m}\frac{{\left(1+\frac{1}{v}{\varsigma}^{T}{\mathrm{R}}^{-1}\varsigma\right)}^{-\frac{v+m}{2}}}{\prod_{j=1}^{m}{\left(1+\frac{{\varsigma}_{j}^{2}}{v}\right)}^{-\frac{v+1}{2}}},$$

where $${\varsigma}_{j}={t}_{v}^{-1}\left({u}_{j}\right).$$

#### The Archimedean copula

In contrast to the elliptical copula, it is easy to deduce parameterized multivariate distributions from the same class of marginal distributions. Given a function $\phi (x)$ as the generator of the Archimedean copula function, the formula of Archimedean copulas induces a copula by

2.11 $$C \left({u}_{1} {,u}_{2} ,\dots ,{ u}_{m}\right)\equiv {\phi }_{m}\left({\phi }^{-1}\left({u}_{1}\right)+\dots +{\phi }^{-1}\left({u}_{m}\right)\right)$$

Three well-known Archimedean copulas are illustrated below with the following density functions (Table 1).

**Table 1:** The Archimedean copulas

Types	Copula function	Copula multivariate function
Clayton	$\phi \left(t\right)=\frac{1}{\theta }\left({t}^{-\theta }-1\right)$	$C \left({u}_{1} {,u}_{2} ,\dots ,{ u}_{m}\right)={max({{{(u}_{1}}^{-\theta }+\dots +{{u}_{m}}^{-\theta }+m-1)}^{-\frac{1}{\theta }}, 0)}$
Frank	$\phi \left(t\right)=-ln\left(\frac{{exp}\left(-\theta t\right)-1}{{exp}\left(-t\right)-1}\right)$	$C \left({u}_{1} {,u}_{2} ,\dots ,{ u}_{m}\right)=-ln\left(1+\frac{({exp}\left(-\theta {u}_{1}\right)-1)\dots ({exp}\left(-\theta {u}_{m}\right)-1)}{{exp}\left(-\theta \right)-1}\right)$
Gumbel	$\phi \left(t\right)={(-lnt)}^{\theta }$	$C \left({u}_{1} {,u}_{2} ,\dots ,{ u}_{m}\right)={exp}\left(-{\left({\left(-ln{u}_{1}\right)}^{\theta }+\dots +{(-ln{u}_{m})}^{\theta }\right)}^{\frac{1}{\theta }}\right)$

Although the Archimedean copula requires only one parameter in the estimation, the partial distribution function is not easy to calculate in high dimensions for the joint density function. Thus, we choose the MGC to build up the joint distribution in “Stressed portfolio optimization and its scaling effect” section for ease of computation.

## The semiparametric method

The semiparametric method combines the nonparametric kernel and the parametric copula methods to describe the marginal distribution of each underlying asset and the joint distribution of the portfolio, respectively. Details about the formulation of each nonparametric and parametric method are discussed in the last section. We focus on the estimation procedures described below, including a bias estimation for the optimal bandwidth.

### Optimal bandwidth choice

As mentioned in “Kernel function” section, the choice of bandwidth is not only pivotal as it determines the smoothness of the estimation but also plays a significant role in the weight function on a kernel. In addition, bandwidth choice is a crucial problem in kernel smoothing because no universally accepted approach exists to this problem yet.

One approach of cross-validation theory aims to minimize the mean square error (MSE) between the estimated and true densities. Thus, an appropriate $h$ should determine the degree of smoothness and influence on the MSE between the kernel estimated density ${f}_{\widehat{p}}\left(x\right)$ and its true density ${f}_{p}\left(x\right)$.

#### Definition 3.1

The variance, bias, and MSE of the estimator are defined as

3.1 $$\begin{aligned}{\mathrm{Var}}_{p}({f}_{\widehat{p}}(X))&={E}_{p}{[{f}_{\widehat{p}}\left(X\right)-{E}_{p}[{f}_{\widehat{p}}\left(X\right)]]}^{2},\\{\mathrm{Bias}}_{p}({f}_{\widehat{p}}(X))&={E}_{p}\left[{f}_{\widehat{p}}\left(X\right)\right]-{f}_{p}\left(x\right),\\ {\mathrm{MSE}}_{p}\left({f}_{\widehat{p}}\left(X\right)\right)&={E}_{p}{\left[{f}_{\widehat{p}}\left(X\right)-{f}_{p}\left(X\right)\right]}^{2}.\end{aligned}$$

Similarly, we could get results

$${\mathrm{MSE}}_{p}\left({f}_{\widehat{p}}(X)\right)={\mathrm{Var}}_{p}\left({f}_{\widehat{p}}(X)\right)+{{\mathrm{Bias}}_{p}}^{2}\left({f}_{\widehat{p}}(X)\right).$$

Let the density function ${f}_{\widehat{p}}(X)$ bound second derivative ${f}_{\widehat{p}}^{\prime\prime}\left(X\right)$, leading to Taylor expansion,

3.2 $${\mathrm{Bias}}_{p}\left({f}_{\widehat{p}}\left(X\right)\right)=\frac{1}{2}{h}^{2}{f}_{\widehat{p}}^{\prime\prime}\left(x\right){k}_{2}+o\left({h}^{2}\right),$$

3.3 $${\mathrm{Var}}_{p}\left({f}_{\widehat{p}}\left(X\right)\right)=\frac{{f}_{\widehat{p}}\left(X\right){k}_{1}}{nh}+o\left(\frac{1}{nh}\right),$$

where ${k}_{1}=\int {K}^{2}\left(u\right)du,$ ${k}_{2}=\int {u}^{2}K\left(u\right)du.$ See Horová et al. (2012) for details.

From (3.2) and (3.3), we derive the MSE of the kernel density estimators as

$${\mathrm{MSE}}_{p}\left(x\right)=\frac{s\left({f}_{\widehat{p}}(x)\right)\left[4\int {K}^{2}\left(u\right)du+n{h}^{5}T\left({f}_{\widehat{p}}(x)\right){[\int {u}^{2}K\left(u\right)du]}^{2}\right]}{4nh}+o\left(\frac{1}{nh}\right)+o\left({h}^{4}\right),$$

where $$\mathrm{T}\left({f}_{\widehat{p}}(x)\right)=\int \frac{{{f}_{\widehat{p}}^{\prime\prime}}^{2}\left(x\right)}{{f}_{\widehat{p}}\left(x\right)}dx,$$ $${\mathrm{s}}_{p}\left({f}_{\widehat{p}}(x)\right)={E}_{p}\left[{f}_{\widehat{p}}^{2}\left(x\right)\right].$$

The optimal bandwidth is defined from the truncated ${\mathrm{MSE}}_{p}\left(x\right)$ taking only the first leading order term as,

$${h}_{opt}=\mathrm{arg}\,min\, MSE\left(h\right).$$

In this approach, the optimal bandwidth can be obtained by some straightforward calculations:

3.4 $${h}_{opt}\approx {n}^{-\frac{1}{5}}{{k}_{1}}^\frac{1}{5}{{k}_{2}}^{-\frac{2}{5}}{\left[\mathrm{T}\left({f}_{\widehat{p}}\left(x\right)\right)\right]}^{-\frac{1}{5}},$$

where ${k}_{1}=\int {K}^{2}\left(u\right)du,$ ${k}_{2}=\int {u}^{2}K\left(u\right)du.$ For Gaussian kernel ${k}_{1}=\sqrt{\frac{1}{4\pi }} ,{k}_{2}=1$; therefore,

3.5 $${h}_{opt}={n}^{-\frac{1}{5}}{\left(4\pi \right)}^{-\frac{1}{10}}{\left[\mathrm{T}\left({f}_{\widehat{p}}\left(x\right)\right)\right]}^{-\frac{1}{5}}.$$

#### Bias estimation for the perturbed optimal bandwidth

Here, we provide a perturbation analysis and show that the error of the Gaussian kernel function deviating from the optimal bandwidth is uniformly bounded.

##### Lemma 3.2

*Given the Gaussian kernel function* ${K}_{{h}_{opt}}\left(t\right)=\frac{1}{{h}_{opt}}K\left(\frac{t}{{h}_{opt}}\right)$ *with the optimal bandwidth choice* ${h}_{opt}>0,$ *for any estimation error* $\upvarepsilon >0$, *there exists an independent constant* $M,$ *such that* $\left|{K}_{{h}_{opt}}\left(t\right)- {K}_{{h}_{opt}+\upvarepsilon }\left(t\right)\right|<M\upvarepsilon$, for $t\in R$.

*This means that the bias between the optimal kernel and its perturbed density is uniformly bounded.*

##### *Proof*

Use Taylor expansion and the uniformly bounded property for the normal density.

Introducing the telescope expression, we obtain

$$\begin{aligned}\left|{K}_{{h}_{opt}}\left(t\right)- {K}_{{h}_{opt}+\upvarepsilon }\left(t\right)\right|\\ \quad &=\left|\frac{1}{{h}_{opt}}K\left(\frac{t}{{h}_{opt}}\right)-\frac{1}{{h}_{opt}+\upvarepsilon }K\left(\frac{t}{{h}_{opt}}\right)+\frac{1}{{h}_{opt}+\upvarepsilon }K\left(\frac{t}{{h}_{opt}}\right)-\frac{1}{{h}_{opt}+\upvarepsilon }K\left(\frac{t}{{h}_{opt}+\upvarepsilon }\right)\right|\\ &\quad\le \left|\left(\frac{1}{{h}_{opt}}-\frac{1}{{h}_{opt}+\upvarepsilon }\right)K\left(\frac{t}{{h}_{opt}}\right)\right|+\left|\frac{1}{{h}_{opt}+\upvarepsilon }\left(K\left(\frac{t}{{h}_{opt}}\right)-K\left(\frac{t}{{h}_{opt}+\upvarepsilon }\right)\right)\right|.\end{aligned}$$

The first term on the left is bounded by ${M}_{1}\upvarepsilon$ regardless of the variable $t$ for some independent constant ${M}_{1}$. Because the Gaussian kernel function is a normal density function, by the mean-value theorem, the second term on the right is bounded above by ${M}_{2}\upvarepsilon$ for some independent constant ${M}_{2}.$ Therefore, $\left|{K}_{{h}_{opt}}\left(t\right)- {K}_{{h}_{opt}+\upvarepsilon }\left(t\right)\right|\le \left({M}_{1}+{M}_{2}\right)\upvarepsilon$ for an arbitrary $t$ is obtained. □

### The joint distribution of portfolio

As a semiparametric estimation, it has nonparametric and parametric components. The kernel method offers the marginal distribution of each asset under nonparametric estimation, and the copula method is common in parametric estimation, which builds up the joint distribution between marginal distributions. After combining these two components, the joint distribution of the portfolio is obtained.

#### Definition 3.3

The joint distribution of assets in our portfolio is as given below:

3.6 $$f\left({x}_{1},{x}_{2},\dots {x}_{n}\right)=c\left({F}_{1}({x}_{1}),{{F}_{2}(x}_{2}),\dots {{F}_{n}(x}_{n})\right)\prod_{i=1}^{n}{f}_{i}\left({x}_{i}\right),$$

where $c\left({x}_{1},{x}_{2},\dots {x}_{n}\right)$ are copulas using parametric methods, and ${f}_{i}\left({x}_{i}\right)$ is the marginal distribution using nonparametric methods.

Once the joint distribution for the multivariate $\left({X}_{1},\dots ,{X}_{n}\right)$ is estimated, its portfolio $P$ with different weights $({w}_{1},..,{w}_{n}$) is defined by,

3.7 $$P\left({X}_{1},\dots ,{X}_{n,}{w}_{1},..,{w}_{n}\right)=\sum_{i=1}^{n}{w}_{i}{X}_{i},$$

where ${w}_{i}$ and ${X}_{i,}$ are the weight and value of ${i}{th}$ asset, respectively. The total sum $\sum_{i=1}^{n}{w}_{i}=1$. When a weight ${w}_{i}$ is nonnegative, it means that the corresponding asset is not allowed for short selling.

#### Parameter estimation

Maximum likelihood estimation (MLE) was employed to estimate model parameters. Based on the joint density function,

3.8 $$f\left({x}_{1},{x}_{2}\dots ,{x}_{n}\right)=c\left({F}_{1}\left({x}_{1}\right),{F}_{2}\left({x}_{2}\right)\dots {F}_{n}\left({x}_{n}\right)\right)\prod_{i=1}^{n}{f}_{i}\left({x}_{i}\right),$$

where $c\left({x}_{1},{x}_{2}\dots {x}_{n}\right)=\frac{{\partial }^{n}C({x}_{1},{x}_{2}\dots {x}_{n})}{\partial {x}_{1}\partial {x}_{2}\dots \partial {x}_{n}}$ is the density of the $n$ dimensional copula $C({x}_{1},{x}_{2}\dots {x}_{n};\theta )$. The log-likelihood function is defined as follows:

3.9 $$L=\sum_{j=1}^{N}logf\left({{x}_{1}}^{\left(j\right)},{{x}_{2}}^{\left(j\right)}\dots {{x}_{n}}^{\left(j\right)}\right)={L}_{C}+\sum_{i=1}^{n}{L}_{i}$$

where ${\mathrm{L}}_{C}=\sum_{j=1}^{N}log c({F}_{1}^{\left(j\right)},{F}_{2}^{\left(j\right)}\dots {F}_{n}^{\left(j\right)})$ is the log-likelihood function from the independent term with the copula $C$ function, the rest term ${L}_{i}=\sum_{j=1}^{N}log{f}_{j}({{x}_{i}}^{(j)}), i=\mathrm{1,2}\dots n$ is the log-likelihood function from the dependent term, which is not necessary to estimate parameters using the nonparametric kernel method, where $log$ denotes the natural logarithm. Thus, only parameters in ${\mathrm{L}}_{C}$ need to be estimated. Let $\theta$ denote the parameter set of copula $C$. This can be estimated by the following full MLE:

3.10 $$\widehat{\theta }={arg}\,{max}_{\theta }{L}_{C}\left(\theta \right)={arg}\,{max}_{\theta }\sum_{j=1}^{N}logc\left({F}_{1}\left({x}_{1}^{\left(j\right)}\right),{F}_{2}\left({x}_{2}^{\left(j\right)}\right)\dots {F}_{n}\left({x}_{n}^{\left(j\right)}\right);\theta \right).$$

## Stressed portfolio optimization and its scaling effect

This section introduces the methodology for stressed portfolio optimization, which includes specific procedures for constructing an optimal portfolio under tail risk and its scaling effect. We extend the use of risk measures (Artzner et al. 1999) for portfolio optimization using the previously mentioned semiparametric method. The optimal scales of such stressed portfolios are studied by maximizing risk-sensitive value measures (Miyahara 2010).

### Risk measure minimization for stressed portfolio

As a regulatory standard or internal control for financial institutions, risk measures provide extreme information about potential value losses. Owing to its simplicity and clarification in risk management, VaR is the most conventional measure to estimate the loss of asset value, given a certain confidence level; therefore, an adequate capital amount is gauged to prevent negative impacts.

#### Definition 4.1

$V{aR}_{\alpha }$ is defined as a quantile in statistics:

4.1 $$V{aR}_{\alpha }\left(X\right)={inf}\left\{l\in R:P\left(X>l\right)\le 1-\alpha \right\}$$

where $\alpha$ is the confidence level, and $X$ denotes either the loss of asset value or its loss return.

Conditional value-at-risk (CVaR), also known as expected shortfall, is a stringent risk assessment used to estimate the average losses exceeding VaR.

#### Definition 4.2

${CVaR}_{\mathrm{\alpha }}$ is defined as a conditional expectation:

$${CVaR}_{\mathrm{\alpha }}(X)=E\left(X|X\ge {VaR}_{\alpha }(X)\right),$$

where $\alpha$ is the confidence level, the variable $X$ represents the loss value or its return, and ${VaR}_{\alpha }(X)$ is defined above.

Note that both values of ${VaR}_{\mathrm{\alpha }}$ and ${CVaR}_{\mathrm{\alpha }}$ are variable $X$ dependent. This means that they are not constant, even though the value of $\alpha$ is given. When the variable $X$ is a portfolio, such as $P$ defined in Eq. (3.7), minimizing nonlinear risk measures such as ${VaR}_{\mathrm{\alpha }}$ and ${CVaR}_{\mathrm{\alpha }}$ over the feasible set of portfolio weights, possibly in high dimensions, must be solved numerically. Discussions on data analysis and computational schemes are presented in “Statistical estimation for semiparametric method” section.

### Value measure maximization for the scaling effect

The evaluation of a risk-sensitive portfolio is essential for finance. This section aims to revisit the optimal scale using the risk-sensitive value measures proposed by Miyahara (2010) and discuss some computational issues given stressed portfolios.

#### Definition 4.3

Let $X$ be a linear space of return of portfolio; the risk-sensitive value measure in $X$ is then the following functional defined on $X$:

4.2 $${U}^{\left(\alpha \right)}\left(X\right)=-\frac{1}{\alpha }logE\left({e}^{-\alpha X}\right),$$

where $\alpha$ is the risk aversion parameter and $\alpha \in [\mathrm{0,1}]$.

For a Gaussian multivariate $X$, from its moment generating function

$$E\left({e}^{-\alpha X}\right)={e}^{E\left(-\alpha X\right)+\frac{1}{2}Var\left(-\alpha X\right)}={e}^{-\alpha E\left(X\right)+\frac{{\alpha }^{2}}{2}Var\left(X\right)},$$

the utility function (4.2) is explicitly obtained

$${U}^{\left(\alpha \right)}\left(X\right)=-\frac{1}{\alpha }logE\left({e}^{-\alpha X}\right)=E\left(X\right)-\frac{\alpha }{2}Var\left(X\right):=MV\left(X\right).$$

The mean–variance (MV) value measure is defined above, and the optimal scale for this MV value measure is obtained.

4.3 $${\lambda }_{opt}=\frac{E\left(X\right)}{\alpha Var\left(X\right)},$$

from solving a quadratic minimization over $\lambda$ the scale of portfolio

$$MV\left(\lambda X\right)=E\left(\lambda X\right)-\frac{1}{2}\alpha Var\left(\lambda X\right),=\lambda E\left(X\right)-\frac{1}{2}{\lambda }^{2}\alpha Var\left(X\right).$$

However, when the distribution of $X$ is non-Gaussian, the mean–variance model is the first two leading terms of the risk-sensitive value measure. This can be easily deduced by substituting the Taylor expansion

$${e}^{-\alpha X}=1-\alpha X+\frac{{\alpha }^{2}{X}^{2}}{2}+H.O.T (higher\,order\,terms)$$

into (4.2) and obtain

$$\begin{aligned}{U}^{\left(\alpha \right)}\left(X\right)&=-\frac{1}{\alpha }logE\left({e}^{-\alpha X}\right)\\&=-\frac{1}{\alpha }\mathrm{log}[\mathrm{E}\left(1-\alpha X+\frac{{\alpha }^{2}{X}^{2}}{2}\right)]+H.O.T\\& \approx -\frac{1}{\alpha }E\left(-\alpha X+\frac{{\alpha }^{2}{X}^{2}}{2}\right)+H.O.T\\&=\mathrm{E}\left(\mathrm{X}\right)-\frac{\alpha }{2}E\left({X}^{2}\right)+H.O.T.\end{aligned}$$

If $\mathrm{X}$ is centered at 0, i.e., $\mathrm{E}\left(\mathrm{X}\right)=0$,

$${U}^{\left(\alpha \right)}\left(X\right)\approx MV\left(X\right)+H.O.T.$$

As ${U}^{(\alpha )}\left(\lambda X\right)$ is a concave function of $\lambda$ (Miyahara 2010), the optimal scale of the portfolio can be obtained by maximizing this scaled value measure:

$${U}^{(\alpha )}\left(\lambda X\right)=-\frac{1}{\alpha }logE\left({e}^{-\alpha \lambda X}\right),$$

such that ${\lambda }_{opt}=\frac{{C}_{X}}{\alpha }$, where ${C}_{X}$ is a solution of $E\left({Xe}^{-{C}_{X}X}\right)=0.$

Because our portfolio variable $X$ has a complex structure from the proposed semiparametric method, we adopt the following Monte Carlo estimator to solve the optimal scale as an approximation:

4.4 $${U}^{\left(\alpha \right)}\left(\lambda X\right)\approx -\frac{1}{\alpha }\mathrm{log}\left(\frac{1}{n}\sum_{i=1}^{n}{e}^{-\alpha \lambda {X}^{\left(i\right)}}\right),$$

where $\lambda$ is the scale of the portfolio, $\alpha$ is the risk aversion, $n$ is the sample size, and ${X}^{\left(i\right)}{{}^{\prime}}s$ are random samples from historical simulations.

We comment on the strict concavity of the approximate estimator in (4.4). This can be inherently derived from the concavity of the utility function defined in Eq. (4.2) by taking the random variable $X$ as discrete and uniformly distributed on the set of fixed outcomes $\left\{{X}^{\left(1\right)}, {X}^{\left(2\right)}, \dots ,{X}^{\left(n\right)} \right\}.$ Since the graph of the risk-sensitive value measure over the scale is concave, the peak of this graph is identified as the optimal scale for its associated portfolio.

For investors with different levels of sensitivity to the same risk, we use different values of aversion to calculate the optimal scale. The risk-seeker (0 $<\alpha <0.5$), risk-neutral ($\alpha =0.5$), and risk-averter ($0.5<\alpha <1$) correspond to aversion values of 0.5, 0.5, and 0.5, respectively.

## Empirical studies and data analysis

According to the framework depicted in Sects. Literature review and Stressed portfolio optimization and its scaling effect” sections, we designed the following experiments for stressed portfolio optimization using the semiparametric method. First, we build the marginal distribution for each constituent of the portfolio, given daily data from 2016 to 2020. We then describe the joint distribution of a portfolio with a Gaussian copula, which explains the dependence between these constituents. Second, we solve for the optimal weights from risk measure minimization using the genetic algorithm (GA) within MATLAB’s package. Finally, the optimal scale based on the stressed VaR portfolio is solved numerically using an approximated Monte Carlo estimator. Intensive and heavy computation, which includes modeling by semiparametric estimation and portfolio optimization under tail risk, is executed on a server cluster equipped with four Intel Xeon 5220R CPUs. Each CPU is 2.2 GHz with 24 cores.

### Statistical estimation for semiparametric method

To implement our methodology on real data, we construct a diversified portfolio with five ETFs: Vanguard S&P 500 ETF (VOO), iShares 20 + Year Treasury Bond ETF (TLT), iShares iBoxx investment grade corporate bond ETF (LQD), iShares Gold Trust ETF (IAU), and Vanguard Real Estate Index Fund ETF Shares (VNQ). Daily price data spanning from 2016 to 2020 were retrieved from the Bloomberg database. Daily returns were calculated from the difference between two consecutive log prices.

Our implementation of the optimization models relies on a rolling-window approach. Specifically, at the beginning of each month, we use the return data of the previous three months to calculate the input parameters needed to determine the portfolio weights. Using these weights, we calculate portfolio returns over the next month. The following month, new portfolio weights are determined using updates of the parameter estimates.

The model parameters of the optimal bandwidth for the kernel function and the correlation matrix required in “Literature review” section for our portfolio are time-invariant in each estimate window (three months). The relevant parameters and estimation results are available upon request.

### Optimal weights for risk measure: stressed portfolio optimization

Following the semiparametric model, applications for portfolio optimization under tail risk are presented. Tables 2 and 3 record the empirical results of in-sample fit for a quarterly time span (three months), which is useful for training models. Tables 4 and 5 record the empirical results of out-of-sample fit for a monthly time span, which is useful for testing models.

**Table 2:** The in-sample results of Markowitz model and semiparametric method with VaR

Period	Markowitz model								
	VOO	TLT	LQD	IAU	VNQ	Volatility	Return	SR	VaR
2015.10–2015.12	0.177457	0.236393	0.237593	0.251497	0.097060	0.004914	0.030581	0.306925	0.006247
2015.11–2016.01	0.191533	0.189183	0.330781	0.151453	0.137050	0.002987	− 0.005483	− 0.098757	0.007346
2015.12–2016.02	0.194609	0.355099	0.082842	0.298886	0.068564	0.004341	0.005303	0.056277	0.006773
2016.01–2016.03	0.260998	0.075808	0.533044	0.059611	0.070539	0.002181	0.008187	0.178133	0.003308
2016.02–2016.04	0.438323	0.064790	0.067393	0.369232	0.060262	0.002125	0.035512	0.825654	0.001020
2016.03–2016.05	0.390102	0.240234	0.210918	0.075207	0.083540	0.001978	0.012604	0.308068	0.002448
2016.04–2016.06	0.479150	0.070969	0.089529	0.298833	0.061519	0.002832	0.007287	0.121279	0.004774
2016.05–2016.07	0.738519	0.068578	0.061201	0.072736	0.058966	0.002899	0.016837	0.283215	0.003153
2016.06–2016.08	0.320867	0.055845	0.230660	0.336898	0.055729	0.005984	0.001661	0.010396	0.013003
2016.07–2016.09	0.050000	0.050000	0.050000	0.050000	0.800000	0.006583	0.027021	0.202071	0.011142
2016.08–2016.10	0.139885	0.050013	0.050025	0.710035	0.050042	0.006969	− 0.048987	− 0.354452	0.015765
2016.09–2016.11	0.050087	0.091749	0.050325	0.757647	0.050192	0.003759	− 0.003168	− 0.047676	0.006812
2016.10–2016.12	0.438323	0.064790	0.067393	0.369232	0.060262	0.002109	0.035072	0.821653	0.001010
2016.11–2017.01	0.390102	0.240234	0.210918	0.075207	0.083540	0.001967	0.012639	0.310623	0.002446
2016.12–2017.02	0.479150	0.070969	0.089529	0.298833	0.061519	0.002872	0.007412	0.121802	0.004850
2017.01–2017.03	0.738519	0.068578	0.061201	0.072736	0.058966	0.002898	0.016696	0.280854	0.003139
2017.02–2017.04	0.052075	0.081504	0.053957	0.750060	0.062405	0.005907	0.008514	0.068544	0.010037
2017.03–2017.05	0.050000	0.050000	0.050000	0.050000	0.800000	0.006613	0.027088	0.201664	0.011180
2017.04–2017.06	0.268480	0.506691	0.100368	0.066477	0.057984	0.003795	0.003241	0.037208	0.007439
2017.05–2017.07	0.050087	0.091749	0.050325	0.757647	0.050192	0.003773	− 0.003187	− 0.047763	0.006848
2017.06–2017.08	0.158318	0.147202	0.286875	0.077530	0.330075	0.003091	0.008758	0.134928	0.004218
2017.07–2017.09	0.068086	0.099889	0.581654	0.196896	0.053475	0.002464	0.011891	0.232854	0.003164
2017.08–2017.10	0.054063	0.324698	0.054747	0.226128	0.340363	0.004020	0.052083	0.642620	0.003341
2017.09–2017.11	0.694957	0.056122	0.065574	0.125798	0.057549	0.002685	0.013336	0.240615	0.004102
2017.10–2017.12	0.294399	0.078843	0.166064	0.401023	0.059670	0.003378	0.017698	0.255765	0.005440
2017.11–2018.01	0.050000	0.050000	0.050000	0.050000	0.800000	0.006603	0.026934	0.200794	0.011140
2017.12–2018.02	0.689011	0.050565	0.051537	0.156637	0.052250	0.008303	− 0.012137	− 0.075602	0.015948
2018.01–2018.03	0.050087	0.091749	0.050325	0.757647	0.050192	0.003766	− 0.003195	− 0.047956	0.006847
2018.02–2018.04	0.158318	0.147202	0.286875	0.077530	0.330075	0.003057	0.008730	0.135977	0.004188
2018.03–2018.05	0.068086	0.099889	0.581654	0.196896	0.053475	0.002495	0.011707	0.226234	0.003159
2018.04–2018.06	0.054063	0.324698	0.054747	0.226128	0.340363	0.004048	0.052520	0.643533	0.003367
2018.05–2018.07	0.694957	0.056122	0.065574	0.125798	0.057549	0.002736	0.013147	0.232666	0.004110
2018.06–2018.08	0.325014	0.342751	0.141088	0.076066	0.115081	0.003133	− 0.015913	− 0.260602	0.005865
2018.07–2018.09	0.227873	0.050683	0.242898	0.428362	0.050185	0.005906	0.032595	0.272428	0.009340
2018.08–2018.10	0.050000	0.050000	0.050000	0.800000	0.050000	0.015891	− 0.004267	− 0.014737	0.036630
2018.09–2018.11	0.583894	0.054994	0.249566	0.055026	0.056520	0.006741	0.064711	0.476899	0.006162
2018.10–2018.12	0.158318	0.147202	0.286875	0.077530	0.330075	0.003061	0.008743	0.136008	0.004196
2018.11–2019.01	0.068086	0.099889	0.581654	0.196896	0.053475	0.002444	0.011862	0.234118	0.003148
2018.12–2019.02	0.054063	0.324698	0.054747	0.226128	0.340363	0.004022	0.052921	0.652735	0.003368
2019.01–2019.03	0.694957	0.056122	0.065574	0.125798	0.057549	0.002692	0.013332	0.239855	0.004107
2019.02–2019.04	0.178815	0.345249	0.250347	0.082559	0.143030	0.003362	0.005718	0.078847	0.005336
2019.03–2019.05	0.227873	0.050683	0.242898	0.428362	0.050185	0.005817	0.032549	0.276187	0.009269
2019.04–2019.06	0.050000	0.050000	0.050000	0.800000	0.050000	0.016006	− 0.004323	− 0.014807	0.037009
2019.05–2019.07	0.583894	0.054994	0.249566	0.055026	0.056520	0.006845	0.065511	0.475499	0.006247
2019.06–2019.08	0.059228	0.520589	0.060882	0.063095	0.296206	0.007853	− 0.017620	− 0.114845	0.014456
2019.07–2019.09	0.185526	0.312187	0.109350	0.328851	0.064085	0.003799	− 0.005244	− 0.074500	0.006928
2019.08–2019.10	0.069817	0.050163	0.050104	0.050025	0.779891	0.005226	0.016017	0.149260	0.007558
2019.09–2019.11	0.354102	0.143553	0.259630	0.157578	0.085137	0.002090	0.019192	0.449085	0.001906
2019.10–2019.12	0.450722	0.062094	0.089300	0.325553	0.072330	0.002482	0.026369	0.522830	0.003108
2019.11–2020.01	0.227873	0.050683	0.242898	0.428362	0.050185	0.005841	0.032588	0.275389	0.009291
2019.12–2020.02	0.050000	0.050000	0.050000	0.800000	0.050000	0.015897	− 0.004250	− 0.014678	0.036575
2020.01–2020.03	0.583894	0.054994	0.249566	0.055026	0.056520	0.006741	0.065250	0.480855	0.006186
2020.02–2020.04	0.256336	0.353002	0.113060	0.160268	0.117334	0.007432	0.009408	0.060487	0.011529
2020.03–2020.05	0.400734	0.051429	0.066797	0.429053	0.051987	0.008963	0.001657	0.006922	0.021945
2020.04–2020.06	0.069817	0.050163	0.050104	0.050025	0.779891	0.005254	0.016093	0.149191	0.007597
2020.05–2020.07	0.050059	0.409585	0.243612	0.246698	0.050045	0.007639	− 0.027503	− 0.182747	0.016412
2020.06–2020.08	0.050112	0.050673	0.796835	0.052323	0.050057	0.002337	0.027782	0.585563	0.002510
2020.07–2020.09	0.129021	0.324443	0.074690	0.414853	0.056993	0.009845	− 0.014013	− 0.073286	0.022706
2020.08–2020.10	0.074008	0.425982	0.067759	0.050285	0.381966	0.004700	0.017964	0.186668	0.004731
2020.09–2020.11	0.327937	0.110332	0.315693	0.055792	0.190245	0.003739	0.009645	0.123414	0.006185

Period	Semiparametric method								
	VOO	TLT	LQD	IAU	VNQ	Volatility	Return	SR	VaR
2015.10–2015.12	0.160183	0.201954	0.389901	0.155095	0.092867	0.003968	0.023492	0.290764	0.004389
2015.11–2016.01	0.216752	0.131119	0.448274	0.112298	0.091557	0.002686	− 0.004560	− 0.092644	0.006486
2015.12–2016.02	0.172940	0.343354	0.067331	0.351387	0.064988	0.004277	0.002737	0.027125	0.006675
2016.01–2016.03	0.287010	0.051091	0.560270	0.050384	0.051246	0.002134	0.008876	0.198229	0.002867
2016.02–2016.04	0.530729	0.080792	0.096155	0.230098	0.062226	0.001641	0.035218	1.060395	0.000373
2016.03–2016.05	0.399718	0.218111	0.252014	0.062924	0.067233	0.001865	0.012726	0.329927	0.002181
2016.04–2016.06	0.407809	0.055219	0.058044	0.425151	0.053778	0.002812	0.010829	0.185165	0.003766
2016.05–2016.07	0.687917	0.086119	0.068683	0.096677	0.060604	0.002701	0.016116	0.290572	0.002939
2016.06–2016.08	0.295074	0.058944	0.345485	0.241187	0.059309	0.005659	0.000088	− 0.002902	0.012696
2016.07–2016.09	0.169464	0.063972	0.058819	0.053705	0.654040	0.005967	0.026090	0.215117	0.009871
2016.08–2016.10	0.490535	0.078949	0.127607	0.210662	0.092247	0.003839	− 0.007447	− 0.102409	0.005604
2016.09–2016.11	0.053734	0.255260	0.067222	0.565168	0.058617	0.002900	0.000532	0.001990	0.004256
2016.10–2016.12	0.530729	0.080792	0.096155	0.230098	0.062226	0.001617	0.034387	1.050693	0.000369
2016.11–2017.01	0.399718	0.218111	0.252014	0.062924	0.067233	0.001847	0.012803	0.335297	0.002178
2016.12–2017.02	0.407809	0.055219	0.058044	0.425151	0.053778	0.002863	0.011203	0.188381	0.003825
2017.01–2017.03	0.687917	0.086119	0.068683	0.096677	0.060604	0.002702	0.015835	0.285352	0.002926
2017.02–2017.04	0.059820	0.299233	0.074893	0.404997	0.161057	0.004510	0.009389	0.099466	0.007155
2017.03–2017.05	0.169464	0.063972	0.058819	0.053705	0.654040	0.006025	0.026201	0.213990	0.009905
2017.04–2017.06	0.349012	0.250337	0.257934	0.077485	0.065231	0.003048	0.008530	0.133072	0.005830
2017.05–2017.07	0.053734	0.255260	0.067222	0.565168	0.058617	0.002923	0.000539	0.002094	0.004277
2017.06–2017.08	0.121561	0.082417	0.495424	0.060742	0.239856	0.002705	0.007115	0.123810	0.003664
2017.07–2017.09	0.073791	0.114737	0.509979	0.248497	0.052996	0.002456	0.011837	0.232526	0.003139
2017.08–2017.10	0.074851	0.306618	0.072666	0.167985	0.377880	0.003839	0.048566	0.627128	0.002693
2017.09–2017.11	0.775866	0.052707	0.055846	0.061907	0.053674	0.002652	0.017581	0.323599	0.003499
2017.10–2017.12	0.334962	0.071289	0.272083	0.263743	0.057923	0.002980	0.017407	0.285051	0.005007
2017.11–2018.01	0.169464	0.063972	0.058819	0.053705	0.654040	0.006003	0.025920	0.212413	0.009870
2017.12–2018.02	0.275426	0.065573	0.140699	0.386802	0.131500	0.004183	0.002988	0.030737	0.006914
2018.01–2018.03	0.053734	0.255260	0.067222	0.565168	0.058617	0.002910	0.000541	0.002142	0.004278
2018.02–2018.04	0.121561	0.082417	0.495424	0.060742	0.239856	0.002646	0.007070	0.125743	0.003638
2018.03–2018.05	0.073791	0.114737	0.509979	0.248497	0.052996	0.002485	0.011472	0.222401	0.003134
2018.04–2018.06	0.074851	0.306618	0.072666	0.167985	0.377880	0.003891	0.049404	0.629415	0.002714
2018.05–2018.07	0.775866	0.052707	0.055846	0.061907	0.053674	0.002735	0.017075	0.304586	0.003507
2018.06–2018.08	0.527862	0.270079	0.069391	0.057903	0.074765	0.002891	− 0.011236	− 0.201531	0.004917
2018.07–2018.09	0.163395	0.117267	0.299596	0.369493	0.050250	0.005635	0.026281	0.229476	0.009048
2018.08–2018.10	0.469976	0.153788	0.057899	0.266291	0.052046	0.007470	0.019957	0.130787	0.017045
2018.09–2018.11	0.485838	0.061267	0.326615	0.062098	0.064182	0.005929	0.057045	0.477570	0.005171
2018.10–2018.12	0.121561	0.082417	0.495424	0.060742	0.239856	0.002656	0.007093	0.125695	0.003643
2018.11–2019.01	0.073791	0.114737	0.509979	0.248497	0.052996	0.002434	0.011858	0.234983	0.003122
2018.12–2019.02	0.074851	0.306618	0.072666	0.167985	0.377880	0.003844	0.050094	0.646250	0.002715
2019.01–2019.03	0.775866	0.052707	0.055846	0.061907	0.053674	0.002669	0.017557	0.321148	0.003503
2019.02–2019.04	0.185337	0.143157	0.531074	0.067048	0.073384	0.002593	0.009933	0.183488	0.003530
2019.03–2019.05	0.163395	0.117267	0.299596	0.369493	0.050250	0.005476	0.026232	0.235693	0.008973
2019.04–2019.06	0.469976	0.153788	0.057899	0.266291	0.052046	0.007581	0.020493	0.132420	0.017219
2019.05–2019.07	0.485838	0.061267	0.326615	0.062098	0.064182	0.006122	0.058396	0.473563	0.005243
2019.06–2019.08	0.092558	0.397549	0.119787	0.104515	0.285590	0.006938	− 0.014984	− 0.110994	0.012743
2019.07–2019.09	0.174315	0.320654	0.315521	0.106787	0.082724	0.003515	− 0.009353	− 0.138969	0.006173
2019.08–2019.10	0.402554	0.081189	0.056218	0.051299	0.408739	0.004420	0.019300	0.213619	0.005823
2019.09–2019.11	0.462187	0.133589	0.120307	0.221211	0.062705	0.001887	0.023460	0.610738	0.001066
2019.10–2019.12	0.302124	0.051589	0.053442	0.540133	0.052713	0.002479	0.030260	0.601898	0.002362
2019.11–2020.01	0.163395	0.117267	0.299596	0.369493	0.050250	0.005517	0.026285	0.234431	0.008997
2019.12–2020.02	0.469976	0.153788	0.057899	0.266291	0.052046	0.007489	0.019791	0.129359	0.017014
2020.01–2020.03	0.485838	0.061267	0.326615	0.062098	0.064182	0.005933	0.057920	0.484621	0.005193
2020.02–2020.04	0.272085	0.162933	0.233367	0.201392	0.130223	0.007076	0.017931	0.123754	0.011220
2020.03–2020.05	0.211447	0.054567	0.294013	0.384679	0.055294	0.006421	0.006489	0.047289	0.015313
2020.04–2020.06	0.402554	0.081189	0.056218	0.051299	0.408739	0.004465	0.019502	0.213715	0.005853
2020.05–2020.07	0.062204	0.342805	0.350125	0.186273	0.058592	0.006584	− 0.024562	− 0.189698	0.013904
2020.06–2020.08	0.055680	0.294309	0.415059	0.181089	0.053865	0.002336	0.040450	0.856912	0.001602
2020.07–2020.09	0.201736	0.393465	0.188655	0.144974	0.071170	0.006108	− 0.013680	− 0.115384	0.012968
2020.08–2020.10	0.051031	0.603457	0.050679	0.050169	0.244664	0.004696	0.023206	0.242621	0.004484
2020.09–2020.11	0.225476	0.057092	0.474217	0.051103	0.192112	0.003403	0.006678	0.092000	0.005677

This table reports the optimal weights (five ETFs: VOO, TLT, LQD, IAU, VNQ), volatility (the standard deviation of return in the estimation window), return, Sharpe ratios (adjusted) and VaR of quarterly in-sample for asset allocation portfolios which are constructed using Markowitz model and semiparametric method with VaR ($\alpha =0.05$). All the results are reported for the total sample period (October 2015-November 2020) and estimation window is 3 months

**Table 3:** The in-sample results of Markowitz model and semiparametric method with CVaR

Period	Markowitz model								
	VOO	TLT	LQD	IAU	VNQ	Volatility	Return	SR	CVaR
2015.10–2015.12	0.177457	0.236393	0.237593	0.251497	0.097060	0.004914	0.030581	0.306925	0.006825
2015.11–2016.01	0.191533	0.189183	0.330781	0.151453	0.137050	0.002987	− 0.005483	− 0.098757	0.010544
2015.12–2016.02	0.194609	0.355099	0.082842	0.298886	0.068564	0.004341	0.005303	0.056277	0.007399
2016.01–2016.03	0.260998	0.075808	0.533044	0.059611	0.070539	0.002181	0.008187	0.178133	0.003562
2016.02–2016.04	0.438323	0.064790	0.067393	0.369232	0.060262	0.002125	0.035512	0.825654	0.001101
2016.03–2016.05	0.390102	0.240234	0.210918	0.075207	0.083540	0.001978	0.012604	0.308068	0.002558
2016.04–2016.06	0.479150	0.070969	0.089529	0.298833	0.061519	0.002832	0.007287	0.121279	0.005792
2016.05–2016.07	0.738519	0.068578	0.061201	0.072736	0.058966	0.002899	0.016837	0.283215	0.003518
2016.06–2016.08	0.320867	0.055845	0.230660	0.336898	0.055729	0.005984	0.001661	0.010396	0.014408
2016.07–2016.09	0.050000	0.050000	0.050000	0.050000	0.800000	0.006583	0.027021	0.202071	0.015180
2016.08–2016.10	0.139885	0.050013	0.050025	0.710035	0.050042	0.006969	− 0.048987	− 0.354452	0.016309
2016.09–2016.11	0.050087	0.091749	0.050325	0.757647	0.050192	0.003759	− 0.003168	− 0.047676	0.007419
2016.10–2016.12	0.438323	0.064790	0.067393	0.369232	0.060262	0.002109	0.035072	0.821653	0.001096
2016.11–2017.01	0.390102	0.240234	0.210918	0.075207	0.083540	0.001967	0.012639	0.310623	0.002550
2016.12–2017.02	0.479150	0.070969	0.089529	0.298833	0.061519	0.002872	0.007412	0.121802	0.005819
2017.01–2017.03	0.738519	0.068578	0.061201	0.072736	0.058966	0.002898	0.016696	0.280854	0.003536
2017.02–2017.04	0.052075	0.081504	0.053957	0.750060	0.062405	0.005907	0.008514	0.068544	0.012043
2017.03–2017.05	0.050000	0.050000	0.050000	0.050000	0.800000	0.006613	0.027088	0.201664	0.014977
2017.04–2017.06	0.268480	0.506691	0.100368	0.066477	0.057984	0.003795	0.003241	0.037208	0.007963
2017.05–2017.07	0.050087	0.091749	0.050325	0.757647	0.050192	0.003773	− 0.003187	− 0.047763	0.007451
2017.06–2017.08	0.158318	0.147202	0.286875	0.077530	0.330075	0.003091	0.008758	0.134928	0.004683
2017.07–2017.09	0.068086	0.099889	0.581654	0.196896	0.053475	0.002464	0.011891	0.232854	0.003275
2017.08–2017.10	0.054063	0.324698	0.054747	0.226128	0.340363	0.004020	0.052083	0.642620	0.004368
2017.09–2017.11	0.694957	0.056122	0.065574	0.125798	0.057549	0.002685	0.013336	0.240615	0.004948
2017.10–2017.12	0.294399	0.078843	0.166064	0.401023	0.059670	0.003378	0.017698	0.255765	0.006574
2017.11–2018.01	0.050000	0.050000	0.050000	0.050000	0.800000	0.006603	0.026934	0.200794	0.015075
2017.12–2018.02	0.689011	0.050565	0.051537	0.156637	0.052250	0.008303	− 0.012137	− 0.075602	0.017540
2018.01–2018.03	0.050087	0.091749	0.050325	0.757647	0.050192	0.003766	− 0.003195	− 0.047956	0.007457
2018.02–2018.04	0.158318	0.147202	0.286875	0.077530	0.330075	0.003057	0.008730	0.135977	0.004670
2018.03–2018.05	0.068086	0.099889	0.581654	0.196896	0.053475	0.002495	0.011707	0.226234	0.003285
2018.04–2018.06	0.054063	0.324698	0.054747	0.226128	0.340363	0.004048	0.052520	0.643533	0.004369
2018.05–2018.07	0.694957	0.056122	0.065574	0.125798	0.057549	0.002736	0.013147	0.232666	0.004938
2018.06–2018.08	0.325014	0.342751	0.141088	0.076066	0.115081	0.003133	− 0.015913	− 0.260602	0.005912
2018.07–2018.09	0.227873	0.050683	0.242898	0.428362	0.050185	0.005906	0.032595	0.272428	0.010791
2018.08–2018.10	0.050000	0.050000	0.050000	0.800000	0.050000	0.015891	− 0.004267	− 0.014737	0.045805
2018.09–2018.11	0.583894	0.054994	0.249566	0.055026	0.056520	0.006741	0.064711	0.476899	0.007793
2018.10–2018.12	0.158318	0.147202	0.286875	0.077530	0.330075	0.003061	0.008743	0.136008	0.004663
2018.11–2019.01	0.068086	0.099889	0.581654	0.196896	0.053475	0.002444	0.011862	0.234118	0.003270
2018.12–2019.02	0.054063	0.324698	0.054747	0.226128	0.340363	0.004022	0.052921	0.652735	0.004379
2019.01–2019.03	0.694957	0.056122	0.065574	0.125798	0.057549	0.002692	0.013332	0.239855	0.004960
2019.02–2019.04	0.178815	0.345249	0.250347	0.082559	0.143030	0.003362	0.005718	0.078847	0.006653
2019.03–2019.05	0.227873	0.050683	0.242898	0.428362	0.050185	0.005817	0.032549	0.276187	0.010741
2019.04–2019.06	0.050000	0.050000	0.050000	0.800000	0.050000	0.016006	− 0.004323	− 0.014807	0.045177
2019.05–2019.07	0.583894	0.054994	0.249566	0.055026	0.056520	0.006845	0.065511	0.475499	0.007774
2019.06–2019.08	0.059228	0.520589	0.060882	0.063095	0.296206	0.007853	− 0.017620	− 0.114845	0.015435
2019.07–2019.09	0.185526	0.312187	0.109350	0.328851	0.064085	0.003799	− 0.005244	− 0.074500	0.007017
2019.08–2019.10	0.069817	0.050163	0.050104	0.050025	0.779891	0.005226	0.016017	0.149260	0.007857
2019.09–2019.11	0.354102	0.143553	0.259630	0.157578	0.085137	0.002090	0.019192	0.449085	0.002287
2019.10–2019.12	0.450722	0.062094	0.089300	0.325553	0.072330	0.002482	0.026369	0.522830	0.004027
2019.11–2020.01	0.227873	0.050683	0.242898	0.428362	0.050185	0.005841	0.032588	0.275389	0.010848
2019.12–2020.02	0.050000	0.050000	0.050000	0.800000	0.050000	0.015897	− 0.004250	− 0.014678	0.045159
2020.01–2020.03	0.583894	0.054994	0.249566	0.055026	0.056520	0.006741	0.065250	0.480855	0.007802
2020.02–2020.04	0.256336	0.353002	0.113060	0.160268	0.117334	0.007432	0.009408	0.060487	0.012488
2020.03–2020.05	0.400734	0.051429	0.066797	0.429053	0.051987	0.008963	0.001657	0.006922	0.029802
2020.04–2020.06	0.069817	0.050163	0.050104	0.050025	0.779891	0.005254	0.016093	0.149191	0.007863
2020.05–2020.07	0.050059	0.409585	0.243612	0.246698	0.050045	0.007639	− 0.027503	− 0.182747	0.020880
2020.06–2020.08	0.050112	0.050673	0.796835	0.052323	0.050057	0.002337	0.027782	0.585563	0.002887
2020.07–2020.09	0.129021	0.324443	0.074690	0.414853	0.056993	0.009845	− 0.014013	− 0.073286	0.028485
2020.08–2020.10	0.074008	0.425982	0.067759	0.050285	0.381966	0.004700	0.017964	0.186668	0.004879
2020.09–2020.11	0.327937	0.110332	0.315693	0.055792	0.190245	0.003739	0.009645	0.123414	0.006487

Period	Semiparametric method								
	VOO	TLT	LQD	IAU	VNQ	Volatility	Return	SR	CVaR
2015.10–2015.12	0.170249	0.205401	0.328092	0.209116	0.087142	0.004465	0.027931	0.312809	0.005895
2015.11–2016.01	0.204442	0.162298	0.390541	0.132372	0.110349	0.002855	− 0.005091	− 0.089143	0.009887
2015.12–2016.02	0.144200	0.327415	0.061079	0.404266	0.063040	0.004290	0.005382	0.062733	0.007334
2016.01–2016.03	0.340428	0.061787	0.468457	0.055119	0.074209	0.002139	0.008219	0.192133	0.003532
2016.02–2016.04	0.520584	0.069588	0.074804	0.275540	0.059483	0.001751	0.035719	1.019976	0.000645
2016.03–2016.05	0.416414	0.256692	0.198058	0.062650	0.066186	0.001951	0.013327	0.341491	0.002276
2016.04–2016.06	0.465600	0.061517	0.069158	0.346403	0.057321	0.002826	0.008681	0.153608	0.005411
2016.05–2016.07	0.640602	0.101805	0.079343	0.113891	0.064359	0.002671	0.015308	0.286610	0.003107
2016.06–2016.08	0.309437	0.057180	0.287812	0.288412	0.057158	0.005763	0.000873	0.007576	0.014361
2016.07–2016.09	0.095315	0.050577	0.050341	0.050158	0.753609	0.006504	0.027058	0.208007	0.014738
2016.08–2016.10	0.569637	0.060120	0.079174	0.223522	0.067546	0.003957	− 0.002269	− 0.028669	0.006309
2016.09–2016.11	0.050167	0.226937	0.050736	0.621761	0.050400	0.003122	− 0.000411	− 0.006588	0.005056
2016.10–2016.12	0.520584	0.069588	0.074804	0.275540	0.059483	0.001746	0.035515	1.017142	0.000643
2016.11–2017.01	0.416414	0.256692	0.198058	0.062650	0.066186	0.001948	0.013273	0.340702	0.002269
2016.12–2017.02	0.465600	0.061517	0.069158	0.346403	0.057321	0.002790	0.008688	0.155731	0.005439
2017.01–2017.03	0.640602	0.101805	0.079343	0.113891	0.064359	0.002668	0.015480	0.290127	0.003122
2017.02–2017.04	0.054454	0.212083	0.059020	0.567892	0.106551	0.005153	0.009406	0.091264	0.009831
2017.03–2017.05	0.095315	0.050577	0.050341	0.050158	0.753609	0.006399	0.026786	0.209292	0.014545
2017.04–2017.06	0.354142	0.368440	0.150845	0.067697	0.058877	0.003374	0.006581	0.097524	0.007739
2017.05–2017.07	0.050167	0.226937	0.050736	0.621761	0.050400	0.003119	− 0.000415	− 0.006651	0.005076
2017.06–2017.08	0.123930	0.085716	0.443321	0.060062	0.286971	0.002794	0.007694	0.137678	0.004084
2017.07–2017.09	0.069641	0.083259	0.643010	0.150291	0.053800	0.002437	0.011298	0.231797	0.003266
2017.08–2017.10	0.102852	0.282229	0.101506	0.180141	0.333273	0.003822	0.047058	0.615671	0.002837
2017.09–2017.11	0.727687	0.050349	0.051084	0.120469	0.050410	0.002671	0.014209	0.265971	0.004631
2017.10–2017.12	0.319734	0.067851	0.235020	0.321115	0.056280	0.003085	0.017981	0.291406	0.006253
2017.11–2018.01	0.095315	0.050577	0.050341	0.050158	0.753609	0.006501	0.026705	0.205402	0.014638
2017.12–2018.02	0.381969	0.053885	0.062446	0.432545	0.069155	0.005350	0.002117	0.019789	0.010982
2018.01–2018.03	0.050167	0.226937	0.050736	0.621761	0.050400	0.003134	− 0.000414	− 0.006597	0.005081
2018.02–2018.04	0.123930	0.085716	0.443321	0.060062	0.286971	0.002771	0.007715	0.139189	0.004073
2018.03–2018.05	0.069641	0.083259	0.643010	0.150291	0.053800	0.002444	0.011332	0.231808	0.003276
2018.04–2018.06	0.102852	0.282229	0.101506	0.180141	0.333273	0.003831	0.046945	0.612694	0.002837
2018.05–2018.07	0.727687	0.050349	0.051084	0.120469	0.050410	0.002695	0.014238	0.264126	0.004621
2018.06–2018.08	0.377315	0.378339	0.095375	0.061079	0.087891	0.003044	− 0.015549	− 0.255371	0.005703
2018.07–2018.09	0.169688	0.058765	0.315583	0.405745	0.050219	0.005605	0.030132	0.268782	0.010706
2018.08–2018.10	0.497298	0.094449	0.057233	0.299211	0.051809	0.008162	0.025253	0.154706	0.022619
2018.09–2018.11	0.532304	0.055444	0.299041	0.055714	0.057497	0.006445	0.061374	0.476160	0.007083
2018.10–2018.12	0.123930	0.085716	0.443321	0.060062	0.286971	0.002769	0.007700	0.139067	0.004067
2018.11–2019.01	0.069641	0.083259	0.643010	0.150291	0.053800	0.002440	0.011256	0.230646	0.003262
2018.12–2019.02	0.102852	0.282229	0.101506	0.180141	0.333273	0.003826	0.047216	0.617051	0.002843
2019.01–2019.03	0.727687	0.050349	0.051084	0.120469	0.050410	0.002605	0.014307	0.274608	0.004641
2019.02–2019.04	0.191454	0.244370	0.411765	0.062929	0.089481	0.002911	0.008499	0.146005	0.005079
2019.03–2019.05	0.169688	0.058765	0.315583	0.405745	0.050219	0.005647	0.030029	0.265866	0.010727
2019.04–2019.06	0.497298	0.094449	0.057233	0.299211	0.051809	0.008078	0.024824	0.153660	0.022310
2019.05–2019.07	0.532304	0.055444	0.299041	0.055714	0.057497	0.006451	0.060973	0.472618	0.007063
2019.06–2019.08	0.069054	0.453305	0.077644	0.076032	0.323964	0.007402	− 0.016259	− 0.109829	0.014525
2019.07–2019.09	0.201803	0.343005	0.176895	0.206749	0.071548	0.003705	− 0.007932	− 0.107061	0.006596
2019.08–2019.10	0.271932	0.056464	0.052748	0.050796	0.568061	0.004720	0.018631	0.197376	0.007786
2019.09–2019.11	0.599527	0.061662	0.057349	0.226609	0.054852	0.002024	0.028534	0.705009	0.001417
2019.10–2019.12	0.433636	0.055530	0.063960	0.387126	0.059748	0.002460	0.027248	0.553920	0.003397
2019.11–2020.01	0.169688	0.058765	0.315583	0.405745	0.050219	0.005694	0.030171	0.264953	0.010796
2019.12–2020.02	0.497298	0.094449	0.057233	0.299211	0.051809	0.008046	0.024914	0.154823	0.022307
2020.01–2020.03	0.532304	0.055444	0.299041	0.055714	0.057497	0.006379	0.062149	0.487173	0.007091
2020.02–2020.04	0.334122	0.265563	0.117092	0.192897	0.090327	0.006448	0.014887	0.115437	0.011486
2020.03–2020.05	0.296114	0.050327	0.144534	0.458646	0.050379	0.007604	0.004694	0.030868	0.024710
2020.04–2020.06	0.271932	0.056464	0.052748	0.050796	0.568061	0.004748	0.018819	0.198164	0.007849
2020.05–2020.07	0.054594	0.352759	0.335639	0.204413	0.052594	0.006918	− 0.025675	− 0.185569	0.018545
2020.06–2020.08	0.051226	0.171718	0.545539	0.180761	0.050756	0.002180	0.039226	0.899665	0.001814
2020.07–2020.09	0.185030	0.386198	0.100052	0.267220	0.061501	0.007775	− 0.013058	− 0.083976	0.021912
2020.08–2020.10	0.096852	0.391626	0.101912	0.050289	0.359321	0.003838	0.017604	0.229317	0.004617
2020.09–2020.11	0.153510	0.108050	0.461168	0.059915	0.217357	0.003363	0.004570	0.067949	0.005794

This table reports the optimal weights (five ETFs: VOO, TLT, LQD, IAU, VNQ), volatility (the standard deviation of return in the estimation window), return, Sharpe ratios (adjusted) and CVaR of quarterly in-sample for asset allocation portfolios which are constructed using Markowitz model and semiparametric method with CVaR ($\alpha =0.05$). All the results are reported for the total sample period (October 2015-November 2020) and estimation window is 3 months

**Table 4:** The out-of-sample results of Markowitz model and semiparametric method with VaR

Period	Markowitz model								
	VOO	TLT	LQD	IAU	VNQ	Volatility	Return	SR	VaR
2016.01	0.177457	0.236393	0.237593	0.251497	0.097060	0.004943	0.031138	0.310756	0.006319
2016.02	0.191533	0.189183	0.330781	0.151453	0.137050	0.003019	− 0.005552	− 0.098852	0.007427
2016.03	0.194609	0.355099	0.082842	0.298886	0.068564	0.004389	0.005317	0.055825	0.006111
2016.04	0.260998	0.075808	0.533044	0.054747	0.070539	0.002226	0.008347	0.178130	0.003372
2016.05	0.438323	0.064790	0.067393	0.369232	0.060262	0.002141	0.035697	0.823922	0.001026
2016.06	0.390102	0.240234	0.210918	0.075207	0.083540	0.002005	0.012844	0.309909	0.002488
2016.07	0.479150	0.070969	0.089529	0.298833	0.061519	0.002885	0.007432	0.121583	0.004865
2016.08	0.738519	0.068578	0.061201	0.072736	0.054747	0.002956	0.016902	0.278845	0.003187
2016.09	0.320867	0.054747	0.230660	0.336898	0.054747	0.006014	0.001688	0.010570	0.013133
2016.10	0.054747	0.054747	0.054747	0.054747	0.800000	0.006678	0.027345	0.201620	0.011283
2016.11	0.139885	0.054747	0.054747	0.710035	0.054747	0.006985	− 0.049834	− 0.359704	0.015914
2016.12	0.054747	0.091749	0.054747	0.757647	0.054747	0.003830	− 0.003225	− 0.047541	0.006936
2017.01	0.438323	0.064790	0.067393	0.369232	0.060262	0.002141	0.035697	0.823922	0.001026
2017.02	0.390102	0.240234	0.210918	0.075207	0.083540	0.002005	0.012844	0.309909	0.002488
2017.03	0.479150	0.070969	0.089529	0.298833	0.061519	0.002885	0.007432	0.121583	0.004865
2017.04	0.738519	0.068578	0.061201	0.072736	0.054747	0.002956	0.016902	0.278845	0.003187
2017.05	0.054747	0.081504	0.054747	0.750060	0.062405	0.005917	0.008666	0.069709	0.010132
2017.06	0.054747	0.054747	0.054747	0.054747	0.800000	0.006678	0.027345	0.201620	0.011283
2017.07	0.268480	0.506691	0.100368	0.066477	0.054747	0.003863	0.003252	0.036699	0.007514
2017.08	0.054747	0.091749	0.054747	0.757647	0.054747	0.003830	− 0.003225	− 0.047541	0.006936
2017.09	0.158318	0.147202	0.286875	0.077530	0.330075	0.003094	0.008857	0.136398	0.004243
2017.10	0.068086	0.099889	0.581654	0.196896	0.054747	0.002497	0.011911	0.230163	0.003186
2017.11	0.054747	0.324698	0.054747	0.226128	0.340363	0.004058	0.054747	0.669423	0.003384
2017.12	0.694957	0.054747	0.065574	0.125798	0.054747	0.002740	0.013373	0.236429	0.004147
2018.01	0.294399	0.078843	0.166064	0.401023	0.054747	0.003388	0.017857	0.257384	0.005469
2018.02	0.054747	0.054747	0.054747	0.054747	0.800000	0.006678	0.027345	0.201620	0.011283
2018.03	0.689011	0.054747	0.054747	0.156637	0.054747	0.008469	− 0.012166	− 0.074287	0.016114
2018.04	0.054747	0.091749	0.054747	0.757647	0.054747	0.003830	− 0.003225	− 0.047541	0.006936
2018.05	0.158318	0.147202	0.286875	0.077530	0.330075	0.003094	0.008857	0.136398	0.004243
2018.06	0.068086	0.099889	0.581654	0.196896	0.054747	0.002497	0.011911	0.230163	0.003186
2018.07	0.054747	0.324698	0.054747	0.226128	0.340363	0.004058	0.054747	0.669423	0.003384
2018.08	0.694957	0.054747	0.065574	0.125798	0.054747	0.002740	0.013373	0.236429	0.004147
2018.09	0.325014	0.342751	0.141088	0.076066	0.115081	0.003169	− 0.016129	− 0.261055	0.005934
2018.10	0.227873	0.054747	0.242898	0.428362	0.054747	0.005928	0.033072	0.275433	0.009425
2018.11	0.054747	0.054747	0.054747	0.800000	0.054747	0.016173	− 0.004332	− 0.014681	0.037222
2018.12	0.583894	0.054747	0.249566	0.054747	0.054747	0.006862	0.065737	0.475957	0.006263
2019.01	0.158318	0.147202	0.286875	0.077530	0.330075	0.003094	0.008857	0.136398	0.004243
2019.02	0.068086	0.099889	0.581654	0.196896	0.054747	0.002497	0.011911	0.230163	0.003186
2019.03	0.054747	0.324698	0.054747	0.226128	0.340363	0.004058	0.054747	0.669423	0.003384
2019.04	0.694957	0.054747	0.065574	0.125798	0.054747	0.002740	0.013373	0.236429	0.004147
2019.05	0.178815	0.345249	0.250347	0.082559	0.143030	0.003428	0.005799	0.078505	0.005423
2019.06	0.227873	0.054747	0.242898	0.428362	0.054747	0.005928	0.033072	0.275433	0.009425
2019.07	0.054747	0.054747	0.054747	0.800000	0.054747	0.016173	− 0.004332	− 0.014681	0.037222
2019.08	0.583894	0.054747	0.249566	0.054747	0.054747	0.006862	0.065737	0.475957	0.006263
2019.09	0.054747	0.520589	0.060882	0.063095	0.296206	0.007866	− 0.017978	− 0.116925	0.014608
2019.10	0.185526	0.312187	0.109350	0.328851	0.064085	0.003843	− 0.005305	− 0.074443	0.007008
2019.11	0.069817	0.054747	0.054747	0.054747	0.779891	0.005293	0.016356	0.150570	0.007682
2019.12	0.354102	0.143553	0.259630	0.157578	0.085137	0.002122	0.019442	0.448288	0.001931
2020.01	0.450722	0.062094	0.089300	0.325553	0.072330	0.002505	0.026534	0.521304	0.003131
2020.02	0.227873	0.054747	0.242898	0.428362	0.054747	0.005928	0.033072	0.275433	0.009425
2020.03	0.054747	0.054747	0.054747	0.800000	0.054747	0.016173	− 0.004332	− 0.014681	0.037222
2020.04	0.583894	0.054747	0.249566	0.054747	0.054747	0.006862	0.065737	0.475957	0.006263
2020.05	0.256336	0.353002	0.113060	0.160268	0.117334	0.006493	0.009454	0.069593	0.010594
2020.06	0.400734	0.054747	0.066797	0.429053	0.054747	0.009111	0.001678	0.006922	0.022245
2020.07	0.069817	0.054747	0.054747	0.054747	0.779891	0.005293	0.016356	0.150570	0.007682
2020.08	0.054747	0.409585	0.243612	0.246698	0.054747	0.007719	− 0.027772	− 0.182593	0.016567
2020.09	0.054747	0.054747	0.796835	0.054747	0.054747	0.002363	0.028087	0.585492	0.002537
2020.10	0.129021	0.324443	0.074690	0.414853	0.054747	0.009991	− 0.014027	− 0.072283	0.022879
2020.11	0.074008	0.425982	0.067759	0.054747	0.381966	0.004043	0.017995	0.217392	0.004757
2020.12	0.327937	0.110332	0.315693	0.054747	0.190245	0.003744	0.009747	0.124604	0.006219

Period	Semiparametric method								
	VOO	TLT	LQD	IAU	VNQ	Volatility	Return	SR	VaR
2016.01	0.160183	0.201954	0.389901	0.155095	0.092867	0.004933	0.031204	0.312025	0.006300
2016.02	0.216752	0.131119	0.448274	0.112298	0.091557	0.003016	− 0.005562	− 0.099114	0.007419
2016.03	0.172940	0.343354	0.067331	0.351387	0.064988	0.004394	0.005335	0.055969	0.006118
2016.04	0.287010	0.054747	0.560270	0.054747	0.054747	0.002226	0.008351	0.178215	0.003367
2016.05	0.530729	0.080792	0.096155	0.230098	0.062226	0.002136	0.035733	0.826709	0.001019
2016.06	0.399718	0.218111	0.252014	0.062924	0.067233	0.002004	0.012855	0.310363	0.002485
2016.07	0.407809	0.054747	0.054747	0.425151	0.054747	0.002885	0.007420	0.121381	0.004855
2016.08	0.687917	0.086119	0.068683	0.096677	0.060604	0.002954	0.016922	0.279345	0.003185
2016.09	0.295074	0.054747	0.345485	0.241187	0.054747	0.006011	0.001698	0.010658	0.013130
2016.10	0.169464	0.063972	0.054747	0.054747	0.654040	0.006672	0.027376	0.202028	0.011270
2016.11	0.490535	0.078949	0.127607	0.210662	0.092247	0.006954	− 0.050094	− 0.363171	0.015812
2016.12	0.054747	0.255260	0.067222	0.565168	0.054747	0.003822	− 0.003247	− 0.047937	0.006910
2017.01	0.530729	0.080792	0.096155	0.230098	0.062226	0.002136	0.035697	0.825874	0.001019
2017.02	0.399718	0.218111	0.252014	0.062924	0.067233	0.002004	0.012843	0.310043	0.002485
2017.03	0.407809	0.054747	0.054747	0.425151	0.054747	0.002885	0.007413	0.121253	0.004855
2017.04	0.687917	0.086119	0.068683	0.096677	0.060604	0.002954	0.016906	0.279059	0.003185
2017.05	0.054747	0.299233	0.074893	0.404997	0.161057	0.005903	0.008661	0.069835	0.010103
2017.06	0.169464	0.063972	0.054747	0.054747	0.654040	0.006672	0.027349	0.201823	0.011270
2017.07	0.349012	0.250337	0.257934	0.077485	0.065231	0.003856	0.003225	0.036419	0.007498
2017.08	0.054747	0.255260	0.067222	0.565168	0.054747	0.003822	− 0.003244	− 0.047895	0.006910
2017.09	0.121561	0.082417	0.495424	0.060742	0.239856	0.003091	0.008865	0.136680	0.004237
2017.10	0.073791	0.114737	0.509979	0.248497	0.054747	0.002497	0.011912	0.230163	0.003186
2017.11	0.074851	0.306618	0.072666	0.167985	0.377880	0.004056	0.054747	0.669677	0.003378
2017.12	0.775866	0.054747	0.054747	0.061907	0.054747	0.002740	0.013351	0.236031	0.004141
2018.01	0.334962	0.071289	0.272083	0.263743	0.054747	0.003384	0.017858	0.257689	0.005464
2018.02	0.169464	0.063972	0.054747	0.054747	0.654040	0.006672	0.027349	0.201823	0.011270
2018.03	0.275426	0.065573	0.140699	0.386802	0.131500	0.008428	− 0.012242	− 0.075094	0.016023
2018.04	0.054747	0.255260	0.067222	0.565168	0.054747	0.003822	− 0.003244	− 0.047895	0.006910
2018.05	0.121561	0.082417	0.495424	0.060742	0.239856	0.003091	0.008865	0.136680	0.004237
2018.06	0.073791	0.114737	0.509979	0.248497	0.054747	0.002497	0.011912	0.230163	0.003186
2018.07	0.074851	0.306618	0.072666	0.167985	0.377880	0.004056	0.054747	0.669677	0.003378
2018.08	0.775866	0.054747	0.054747	0.061907	0.054747	0.002740	0.013351	0.236031	0.004141
2018.09	0.527862	0.270079	0.069391	0.054747	0.074765	0.003167	− 0.016152	− 0.261580	0.005924
2018.10	0.163395	0.117267	0.299596	0.369493	0.054747	0.005925	0.033102	0.275813	0.009422
2018.11	0.469976	0.153788	0.054747	0.266291	0.054747	0.016089	− 0.004456	− 0.015144	0.037023
2018.12	0.485838	0.061267	0.326615	0.062098	0.064182	0.006855	0.065792	0.476833	0.006253
2019.01	0.121561	0.082417	0.495424	0.060742	0.239856	0.003091	0.008865	0.136680	0.004237
2019.02	0.073791	0.114737	0.509979	0.248497	0.054747	0.002497	0.011912	0.230163	0.003186
2019.03	0.074851	0.306618	0.072666	0.167985	0.377880	0.004056	0.054747	0.669677	0.003378
2019.04	0.775866	0.054747	0.054747	0.061907	0.054747	0.002740	0.013351	0.236031	0.004141
2019.05	0.185337	0.143157	0.531074	0.067048	0.073384	0.003420	0.005777	0.078357	0.005404
2019.06	0.163395	0.117267	0.299596	0.369493	0.054747	0.005925	0.033102	0.275813	0.009422
2019.07	0.469976	0.153788	0.054747	0.266291	0.054747	0.016089	− 0.004456	− 0.015144	0.037023
2019.08	0.485838	0.061267	0.326615	0.062098	0.064182	0.006855	0.065792	0.476833	0.006253
2019.09	0.092558	0.397549	0.119787	0.104515	0.285590	0.007857	− 0.017990	− 0.117138	0.014591
2019.10	0.174315	0.320654	0.315521	0.106787	0.082724	0.003840	− 0.005284	− 0.074225	0.007000
2019.11	0.402554	0.081189	0.054747	0.054747	0.408739	0.005285	0.016337	0.150626	0.007665
2019.12	0.462187	0.133589	0.120307	0.221211	0.062705	0.002120	0.019419	0.448147	0.001923
2020.01	0.302124	0.054747	0.054747	0.540133	0.054747	0.002509	0.026513	0.519973	0.003123
2020.02	0.163395	0.117267	0.299596	0.369493	0.054747	0.005925	0.033102	0.275813	0.009422
2020.03	0.469976	0.153788	0.054747	0.266291	0.054747	0.016089	− 0.004456	− 0.015144	0.037023
2020.04	0.485838	0.061267	0.326615	0.062098	0.064182	0.006855	0.065792	0.476833	0.006253
2020.05	0.272085	0.162933	0.233367	0.201392	0.130223	0.006500	0.009411	0.069192	0.010601
2020.06	0.211447	0.054747	0.294013	0.384679	0.054747	0.009086	0.001653	0.006804	0.022178
2020.07	0.402554	0.081189	0.054747	0.054747	0.408739	0.005285	0.016337	0.150626	0.007665
2020.08	0.062204	0.342805	0.350125	0.186273	0.054747	0.007709	− 0.027786	− 0.182916	0.016542
2020.09	0.054747	0.294309	0.415059	0.181089	0.054747	0.002363	0.028021	0.584064	0.002527
2020.10	0.201736	0.393465	0.188655	0.144974	0.071170	0.009954	− 0.014029	− 0.072564	0.022781
2020.11	0.054747	0.603457	0.054747	0.054747	0.244664	0.004051	0.017969	0.216659	0.004755
2020.12	0.225476	0.054747	0.474217	0.054747	0.192112	0.003741	0.009762	0.124904	0.006214

This table reports the optimal weights (five ETFs: VOO, TLT, LQD, IAU, VNQ), volatility (the standard deviation of return each month), return, Sharpe ratios (adjusted) and VaR of monthly out-of-sample for asset allocation portfolios which is constructed using Markowitz model and semiparametric method with VaR ($\alpha =0.05$). All the results are reported for the total sample period (Janaury 2016-Octomber 2020)

**Table 5:** The summary of out-of-sample results for Markowitz model and semiparametric method with VaR ($\alpha =0.05$)

Year	Markowitz model				Semiparametric method			
	Volatility	Return	SR	VaR	Volatility	Return	SR	VaR
2016	0.063592	0.088099	1.275303	0.006838	0.063507	0.087992	1.275310	0.006822
2017	0.056439	0.197801	3.380649	0.005199	0.056375	0.197725	3.383128	0.005189
2018	0.088482	0.197047	2.147864	0.009467	0.088271	0.196883	2.151138	0.009436
2019	0.084537	0.201679	2.302884	0.008710	0.084367	0.201572	2.306246	0.008685
2020	0.106128	0.162529	1.465489	0.012460	0.105918	0.162289	1.466124	0.012424

According to Eq. (4.1), portfolio VaR is a function of the weight vector $w$ defined by

$${\mathrm{VaR}}_{\alpha }(P\left({\varvec{w}}\right))= g\left({\varvec{w}}\right),$$

where $g$ denotes the function of the weight vector ${\varvec{w}}$, and the optimal weight $\widehat{{\varvec{w}}}$ attains the minimum value of $g\left({\varvec{w}}\right).$ Table 2 records the in-sample fit for the optimal weight vector $\widehat{{\varvec{w}}}$, the performance of each stressed portfolio, and its VaR value for five consecutive years from 2016 to 2020. These performance results, including volatility, return, Sharpe ratio, and VaR, are calculated quarterly.

According to Table 2, although Markowitz’s model and semiparametric method have different objective functions for weight estimation, the two methods have comparable results for the Sharpe ratio. The in-sample results show that the semiparametric method always has a lower VaR than Markowitz’s model.

Similarly, the portfolio CVaR is a function of weight vector $w$ defined by the following equation:

$${\mathrm{CVaR}}_{\alpha }(P\left({\varvec{w}}\right))= k\left({\varvec{w}}\right),$$

where $k$ is a function of the weight vector ${\varvec{w}}$, and the optimal weight $\widehat{{\varvec{w}}}$ is the minimum value of $k\left({\varvec{w}}\right)$. The optimal weight, the performance of each stressed portfolio, and its CVaR value are listed in Table 3.

Tables 2 and 3 demonstrate the in-sample tests of the dataset and the performance measure of the optimal stressed portfolio on a long-term quarterly basis. According to Tables 2 and 3, although Markowitz’s model and semiparametric method have different objective functions for weight estimation, the two methods have comparable results in terms of the Sharpe ratio. The empirical results of the in-sample show that the semiparametric method always has lower VaR and CVaR than Markowitz’s model.

We conduct out-of-sample tests on a short-term monthly basis by using the same set of five ETFs (VOO-equity, TLT-government bond, LQD-corporate bond, IAU-gold, and VNQ-real estate) and compare the performance of portfolios generated from the semiparametric method and Markowitz method from 2016 to 2020, as demonstrated in Table 4.

**Fig. 1:**
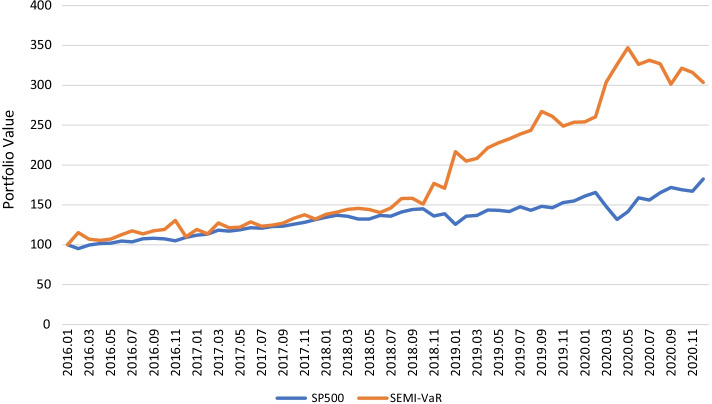
The portfolio value of the semiparametric model with VaR and S&P 500 from 2016 to 2020. This figure shows the portfolio value of S&P 500 (blue line) and Semiparametric method with VaR ($\alpha =0.05, yello line$) in five years (2016 to 2020). The initial value of portfolio is 100. It is clear that the semiparametric method has better performance in return

The results of return, volatility, Sharpe ratio, and risk measures were calculated monthly. As can be seen from Fig. 1, compared to S&P 500, our semiparametric method provides better results in terms of portfolio returns during those five years.

Note that Markowitz’s mean–variance model is profit-oriented. It selects the portfolio with the highest Sharpe ratio from the efficient frontier of the five ETF assets. Nevertheless, the semiparametric method is risk-oriented. Its objective function aims to minimize the VaR/CVaR function. Compared with Markowitz’s mean–variance method, Table 4 is summarized in Table 5. Our semiparametric method reduces the average volatility of the portfolio in those five years and decreases the average return in the same period, simultaneously, but increases the average Sharpe ratio of the portfolio. Our proposed method mitigates not only the whole risk but also the tail risk because our method has a lower portfolio VaR in those five years.

**Fig. 2:**
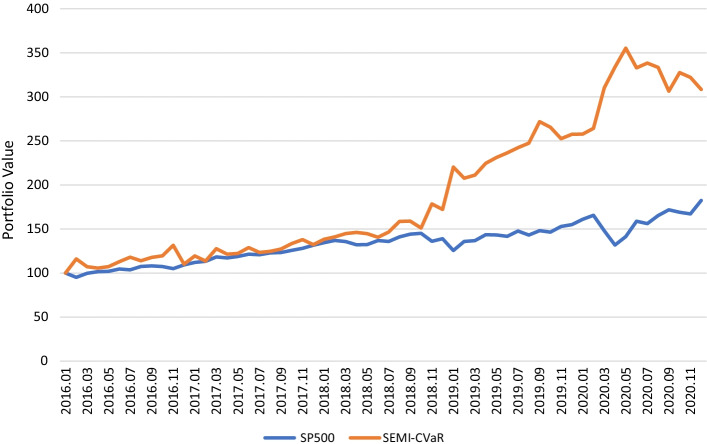
The portfolio value of the semiparametric model with CVaR and S&P 500 from 2016 to 2020. This figure shows the portfolio value of S&P 500 (blue line) and Semiparametric method with CVaR ($\alpha =0.05, yello line$) in five years (2016 to 2020). The initial value of portfolio is 100. It is clear that the semiparametric method has better performance in return

Similarly, the coherent risk measure CVaR is used to compare the results of the semiparametric method and Markowitz’s method within the same test period from 2016 to 2020. Figure 2 depicts the portfolio value of the semiparametric model with CVaR and S&P 500.

As shown in Table 7, which is a summary of Table 6, our semiparametric method reduces average volatility of portfolio in five years, whereas our method decreases average return in the same period. However, the semiparametric method increases the average Sharpe ratio of the portfolio. Our semiparametric method consistently offers better risk management than the Markowitz model in comprehensive risk and tail risk because our method has a lower portfolio CVaR.

**Table 6:** The out-of-sample results of Markowitz model and semiparametric method with CVaR

Period	Markowitz model								
	VOO	TLT	LQD	IAU	VNQ	Volatility	Return	SR	CVaR
2016.01	0.177457	0.236393	0.237593	0.251497	0.097060	0.004943	0.031138	0.310756	0.006841
2016.02	0.191533	0.189183	0.330781	0.151453	0.137050	0.003019	− 0.005552	− 0.098852	0.010590
2016.03	0.194609	0.355099	0.082842	0.298886	0.068564	0.004389	0.005317	0.055825	0.007194
2016.04	0.260998	0.075808	0.533044	0.054747	0.070539	0.002226	0.008347	0.178130	0.003593
2016.05	0.438323	0.064790	0.067393	0.369232	0.060262	0.002141	0.035697	0.823922	0.001111
2016.06	0.390102	0.240234	0.210918	0.075207	0.083540	0.002005	0.012844	0.309909	0.002591
2016.07	0.479150	0.070969	0.089529	0.298833	0.061519	0.002885	0.007432	0.121583	0.005832
2016.08	0.738519	0.068578	0.061201	0.072736	0.054747	0.002956	0.016902	0.278845	0.003560
2016.09	0.320867	0.054747	0.230660	0.336898	0.054747	0.006014	0.001688	0.010570	0.014591
2016.10	0.054747	0.054747	0.054747	0.054747	0.800000	0.006678	0.027345	0.201620	0.015230
2016.11	0.139885	0.054747	0.054747	0.710035	0.054747	0.006985	− 0.049834	− 0.359704	0.016481
2016.12	0.054747	0.091749	0.054747	0.757647	0.054747	0.003830	− 0.003225	− 0.047541	0.007550
2017.01	0.438323	0.064790	0.067393	0.369232	0.060262	0.002141	0.035697	0.823922	0.001111
2017.02	0.390102	0.240234	0.210918	0.075207	0.083540	0.002005	0.012844	0.309909	0.002591
2017.03	0.479150	0.070969	0.089529	0.298833	0.061519	0.002885	0.007432	0.121583	0.005832
2017.04	0.738519	0.068578	0.061201	0.072736	0.054747	0.002956	0.016902	0.278845	0.003560
2017.05	0.054747	0.081504	0.054747	0.750060	0.062405	0.005917	0.008666	0.069709	0.012107
2017.06	0.054747	0.054747	0.054747	0.054747	0.800000	0.006678	0.027345	0.201620	0.015230
2017.07	0.268480	0.506691	0.100368	0.066477	0.054747	0.003863	0.003252	0.036699	0.008100
2017.08	0.054747	0.091749	0.054747	0.757647	0.054747	0.003830	− 0.003225	− 0.047541	0.007550
2017.09	0.158318	0.147202	0.286875	0.077530	0.330075	0.003094	0.008857	0.136398	0.004711
2017.10	0.068086	0.099889	0.581654	0.196896	0.054747	0.002497	0.011911	0.230163	0.003311
2017.11	0.054747	0.324698	0.054747	0.226128	0.340363	0.004058	0.054747	0.669423	0.004411
2017.12	0.694957	0.054747	0.065574	0.125798	0.054747	0.002740	0.013373	0.236429	0.004996
2018.01	0.294399	0.078843	0.166064	0.401023	0.054747	0.003388	0.017857	0.257384	0.006633
2018.02	0.054747	0.054747	0.054747	0.054747	0.800000	0.006678	0.027345	0.201620	0.015230
2018.03	0.689011	0.054747	0.054747	0.156637	0.054747	0.008469	− 0.012166	− 0.074287	0.017673
2018.04	0.054747	0.091749	0.054747	0.757647	0.054747	0.003830	− 0.003225	− 0.047541	0.007550
2018.05	0.158318	0.147202	0.286875	0.077530	0.330075	0.003094	0.008857	0.136398	0.004711
2018.06	0.068086	0.099889	0.581654	0.196896	0.054747	0.002497	0.011911	0.230163	0.003311
2018.07	0.054747	0.324698	0.054747	0.226128	0.340363	0.004058	0.054747	0.669423	0.004411
2018.08	0.694957	0.054747	0.065574	0.125798	0.054747	0.002740	0.013373	0.236429	0.004996
2018.09	0.325014	0.342751	0.141088	0.076066	0.115081	0.003169	− 0.016129	− 0.261055	0.005973
2018.10	0.227873	0.054747	0.242898	0.428362	0.054747	0.005928	0.033072	0.275433	0.010500
2018.11	0.054747	0.054747	0.054747	0.800000	0.054747	0.016173	− 0.004332	− 0.014681	0.045897
2018.12	0.583894	0.054747	0.249566	0.054747	0.054747	0.006862	0.065737	0.475957	0.007856
2019.01	0.158318	0.147202	0.286875	0.077530	0.330075	0.003094	0.008857	0.136398	0.004711
2019.02	0.068086	0.099889	0.581654	0.196896	0.054747	0.002497	0.011911	0.230163	0.003311
2019.03	0.054747	0.324698	0.054747	0.226128	0.340363	0.004058	0.054747	0.669423	0.004411
2019.04	0.694957	0.054747	0.065574	0.125798	0.054747	0.002740	0.013373	0.236429	0.004996
2019.05	0.178815	0.345249	0.250347	0.082559	0.143030	0.003428	0.005799	0.078505	0.006679
2019.06	0.227873	0.054747	0.242898	0.428362	0.054747	0.005928	0.033072	0.275433	0.010500
2019.07	0.054747	0.054747	0.054747	0.800000	0.054747	0.016173	− 0.004332	− 0.014681	0.045897
2019.08	0.583894	0.054747	0.249566	0.054747	0.054747	0.006862	0.065737	0.475957	0.007856
2019.09	0.054747	0.520589	0.060882	0.063095	0.296206	0.007866	− 0.017978	− 0.116925	0.015473
2019.10	0.185526	0.312187	0.109350	0.328851	0.064085	0.003843	− 0.005305	− 0.074443	0.007069
2019.11	0.069817	0.054747	0.054747	0.054747	0.779891	0.005293	0.016356	0.150570	0.007696
2019.12	0.354102	0.143553	0.259630	0.157578	0.085137	0.002122	0.019442	0.448288	0.002312
2020.01	0.450722	0.062094	0.089300	0.325553	0.072330	0.002505	0.026534	0.521304	0.004065
2020.02	0.227873	0.054747	0.242898	0.428362	0.054747	0.005928	0.033072	0.275433	0.010500
2020.03	0.054747	0.054747	0.054747	0.800000	0.054747	0.016173	− 0.004332	− 0.014681	0.045897
2020.04	0.583894	0.054747	0.249566	0.054747	0.054747	0.006862	0.065737	0.475957	0.007856
2020.05	0.256336	0.353002	0.113060	0.160268	0.117334	0.006493	0.009454	0.069593	0.012553
2020.06	0.400734	0.054747	0.066797	0.429053	0.054747	0.009111	0.001678	0.006922	0.030188
2020.07	0.069817	0.054747	0.054747	0.054747	0.779891	0.005293	0.016356	0.150570	0.007696
2020.08	0.054747	0.409585	0.243612	0.246698	0.054747	0.007719	− 0.027772	− 0.182593	0.021088
2020.09	0.054747	0.054747	0.796835	0.054747	0.054747	0.002363	0.028087	0.585492	0.002910
2020.10	0.129021	0.324443	0.074690	0.414853	0.054747	0.009991	− 0.014027	− 0.072283	0.028964
2020.11	0.074008	0.425982	0.067759	0.054747	0.381966	0.004043	0.017995	0.217392	0.004930
2020.12	0.327937	0.110332	0.315693	0.054747	0.190245	0.003744	0.009747	0.124604	0.006512

Period	Semiparametric method								
	VOO	TLT	LQD	IAU	VNQ	Volatility	Return	SR	CVaR
2016.01	0.170249	0.205401	0.328092	0.209116	0.087142	0.004938	0.031142	0.311102	0.006832
2016.02	0.204442	0.162298	0.390541	0.132372	0.110349	0.003017	− 0.005552	− 0.098917	0.010584
2016.03	0.144200	0.327415	0.061079	0.404266	0.063040	0.004399	0.005322	0.055754	0.007197
2016.04	0.340428	0.061787	0.468457	0.055119	0.074209	0.002228	0.008347	0.177975	0.003593
2016.05	0.520584	0.069588	0.074804	0.275540	0.059483	0.002137	0.035696	0.825433	0.001107
2016.06	0.416414	0.256692	0.198058	0.062650	0.066186	0.002004	0.012844	0.309985	0.002588
2016.07	0.465600	0.061517	0.069158	0.346403	0.057321	0.002885	0.007431	0.121570	0.005828
2016.08	0.640602	0.101805	0.079343	0.113891	0.064359	0.002953	0.016903	0.279130	0.003555
2016.09	0.309437	0.057180	0.287812	0.288412	0.057158	0.006012	0.001689	0.010581	0.014591
2016.10	0.095315	0.050577	0.050341	0.050158	0.753609	0.006676	0.027346	0.201685	0.015226
2016.11	0.569637	0.060120	0.079174	0.223522	0.067546	0.006956	− 0.049881	− 0.361546	0.016380
2016.12	0.050167	0.226937	0.050736	0.621761	0.050400	0.003823	− 0.003228	− 0.047662	0.007526
2017.01	0.520584	0.069588	0.074804	0.275540	0.059483	0.002137	0.035668	0.824782	0.001107
2017.02	0.416414	0.256692	0.198058	0.062650	0.066186	0.002004	0.012834	0.309737	0.002588
2017.03	0.465600	0.061517	0.069158	0.346403	0.057321	0.002885	0.007426	0.121486	0.005828
2017.04	0.640602	0.101805	0.079343	0.113891	0.064359	0.002953	0.016888	0.278876	0.003555
2017.05	0.054454	0.212083	0.059020	0.567892	0.106551	0.005909	0.008659	0.069742	0.012084
2017.06	0.095315	0.050577	0.050341	0.050158	0.753609	0.006676	0.027323	0.201517	0.015226
2017.07	0.354142	0.368440	0.150845	0.067697	0.058877	0.003858	0.003249	0.036708	0.008098
2017.08	0.050167	0.226937	0.050736	0.621761	0.050400	0.003823	− 0.003222	− 0.047591	0.007526
2017.09	0.123930	0.085716	0.443321	0.060062	0.286971	0.003092	0.008850	0.136387	0.004705
2017.10	0.069641	0.083259	0.643010	0.150291	0.053800	0.002496	0.011901	0.230041	0.003311
2017.11	0.102852	0.282229	0.101506	0.180141	0.333273	0.004056	0.054705	0.669264	0.004396
2017.12	0.727687	0.050349	0.051084	0.120469	0.050410	0.002740	0.013362	0.236270	0.004992
2018.01	0.319734	0.067851	0.235020	0.321115	0.056280	0.003385	0.017843	0.257367	0.006629
2018.02	0.095315	0.050577	0.050341	0.050158	0.753609	0.006676	0.027323	0.201517	0.015226
2018.03	0.381969	0.053885	0.062446	0.432545	0.069155	0.008439	− 0.012156	− 0.074495	0.017607
2018.04	0.050167	0.226937	0.050736	0.621761	0.050400	0.003823	− 0.003222	− 0.047591	0.007526
2018.05	0.123930	0.085716	0.443321	0.060062	0.286971	0.003092	0.008850	0.136387	0.004705
2018.06	0.069641	0.083259	0.643010	0.150291	0.053800	0.002496	0.011901	0.230041	0.003311
2018.07	0.102852	0.282229	0.101506	0.180141	0.333273	0.004056	0.054705	0.669264	0.004396
2018.08	0.727687	0.050349	0.051084	0.120469	0.050410	0.002740	0.013362	0.236270	0.004992
2018.09	0.377315	0.378339	0.095375	0.061079	0.087891	0.003168	− 0.016116	− 0.260928	0.005971
2018.10	0.169688	0.058765	0.315583	0.405745	0.050219	0.005926	0.033046	0.275319	0.010504
2018.11	0.497298	0.094449	0.057233	0.299211	0.051809	0.016093	− 0.004329	− 0.014743	0.045665
2018.12	0.532304	0.055444	0.299041	0.055714	0.057497	0.006858	0.065684	0.475823	0.007849
2019.01	0.123930	0.085716	0.443321	0.060062	0.286971	0.003092	0.008850	0.136387	0.004705
2019.02	0.069641	0.083259	0.643010	0.150291	0.053800	0.002496	0.011901	0.230041	0.003311
2019.03	0.102852	0.282229	0.101506	0.180141	0.333273	0.004056	0.054705	0.669264	0.004396
2019.04	0.727687	0.050349	0.051084	0.120469	0.050410	0.002740	0.013362	0.236270	0.004992
2019.05	0.191454	0.244370	0.411765	0.062929	0.089481	0.003423	0.005794	0.078559	0.006664
2019.06	0.169688	0.058765	0.315583	0.405745	0.050219	0.005926	0.033046	0.275319	0.010504
2019.07	0.497298	0.094449	0.057233	0.299211	0.051809	0.016093	− 0.004329	− 0.014743	0.045665
2019.08	0.532304	0.055444	0.299041	0.055714	0.057497	0.006858	0.065684	0.475823	0.007849
2019.09	0.069054	0.453305	0.077644	0.076032	0.323964	0.007861	− 0.017964	− 0.116903	0.015464
2019.10	0.201803	0.343005	0.176895	0.206749	0.071548	0.003842	− 0.005301	− 0.074417	0.007065
2019.11	0.271932	0.056464	0.052748	0.050796	0.568061	0.005288	0.016343	0.150599	0.007698
2019.12	0.599527	0.061662	0.057349	0.226609	0.054852	0.002121	0.019426	0.448122	0.002303
2020.01	0.433636	0.055530	0.063960	0.387126	0.059748	0.002505	0.026533	0.521248	0.004058
2020.02	0.169688	0.058765	0.315583	0.405745	0.050219	0.005926	0.033075	0.275563	0.010504
2020.03	0.497298	0.094449	0.057233	0.299211	0.051809	0.016093	− 0.004361	− 0.014845	0.045665
2020.04	0.532304	0.055444	0.299041	0.055714	0.057497	0.006858	0.065741	0.476235	0.007849
2020.05	0.334122	0.265563	0.117092	0.192897	0.090327	0.006496	0.009449	0.069517	0.012543
2020.06	0.296114	0.050327	0.144534	0.458646	0.050379	0.009097	0.001675	0.006916	0.030137
2020.07	0.271932	0.056464	0.052748	0.050796	0.568061	0.005288	0.016354	0.150701	0.007698
2020.08	0.054594	0.352759	0.335639	0.204413	0.052594	0.007712	− 0.027774	− 0.182775	0.021065
2020.09	0.051226	0.171718	0.545539	0.180761	0.050756	0.002361	0.028075	0.585659	0.002900
2020.10	0.185030	0.386198	0.100052	0.267220	0.061501	0.009970	− 0.014028	− 0.072441	0.028898
2020.11	0.096852	0.391626	0.101912	0.050289	0.359321	0.004042	0.017995	0.217460	0.004927
2020.12	0.153510	0.108050	0.461168	0.059915	0.217357	0.003741	0.009752	0.124785	0.006505

This table reports the optimal weights (five ETFs: VOO, TLT, LQD, IAU, VNQ), volatility (the standard deviation of return each month), return, Sharpe ratios (adjusted) and CVaR of monthly out-of-sample for asset allocation portfolios which is constructed using Markowitz model and semiparametric method with CVaR ($\alpha =0.05$). All the results are reported for the total sample period (Janaury 2016-Octomber 2020)

**Table 7:** The summary of out-of-sample results for Markowitz model and semiparametric method with CVaR ($\alpha =0.05$)

Year	Markowitz model				Semiparametric method			
	Volatility	Return	SR	CVaR	Volatility	Return	SR	CVaR
2016	0.063592	0.088099	1.275303	0.007930	0.063537	0.088058	1.275770	0.007917
2017	0.056439	0.197801	3.380649	0.006126	0.056394	0.197644	3.380582	0.006118
2018	0.088482	0.197047	2.147864	0.011228	0.088305	0.196891	2.150401	0.011198
2019	0.084537	0.201679	2.302884	0.010076	0.084393	0.201519	2.304930	0.010051
2020	0.106128	0.162529	1.465489	0.015263	0.105947	0.162485	1.467570	0.015229

In addition, we verify the robustness of the semiparametric method in several sensitivity checks. First, we extensively vary the dataset to examine whether our findings are robust with respect to the indices used to represent the asset classes. For example, we add other ETFs or use alternative indices to our portfolio. This procedure often leads to changes in sample size. However, we find that the variation in the dataset does not alter any of our conclusions. Second, we examine whether the performance of our method improves when shorter and longer time series of historical returns are used for parametrization, and we base the estimation method on a rolling-window approach with 2 months and 4 months of historical data available in estimation. We do not observe a consistent improvement in additional tests. Third, we repeat our analysis by utilizing other performance measures. Specifically, we employ the Sortino ratio, which does not change the qualitative nature of our results.

### Optimal scale for value measure: scaling effect

**Fig. 3:**
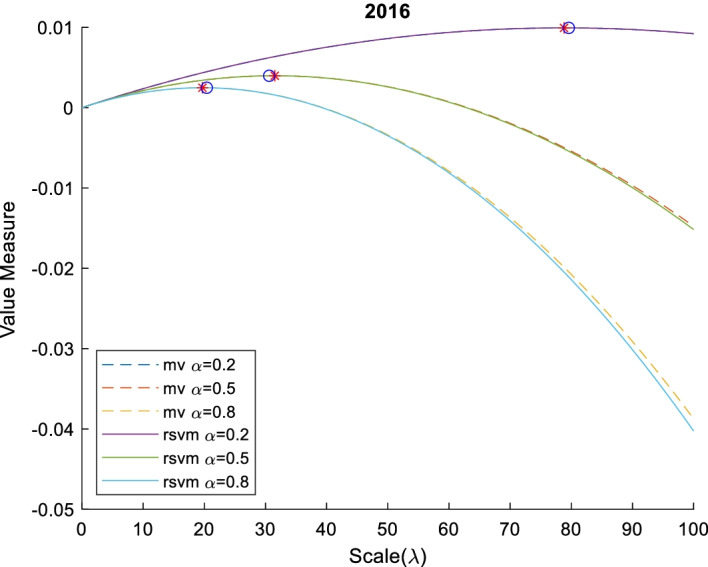
Value measure of RSVM and MV with various risk aversion $\alpha$ and scale $\lambda$ in 2016. This figure shows the relation between value measure (mean–variance model with dash line and risk-sensitive value measure with line) and scale with different investors (risk-averter $\alpha =0.8$, risk-neutral $\alpha =0.5$, and risk-seeker $\alpha =0.2$) in 2016. The red star and blue circle are optimal scale of mean–variance model and risk-sensitive value measure, respectively

**Fig. 4:**
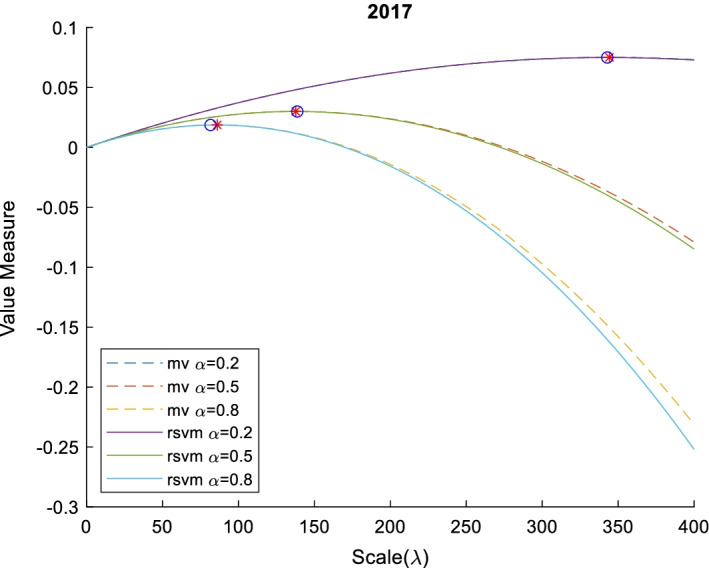
Value measure of RSVM and MV with various risk aversion $\alpha$ and scale $\lambda$ in 2017. This figure shows the relation between value measure (mean–variance model with dash line and risk-sensitive value measure with line) and scale with different investors (risk-averter $\alpha =0.8$, risk-neutral $\alpha =0.5$, and risk-seeker $\alpha =0.2$) in 2017. The red star and blue circle are optimal scale of mean–variance model and risk-sensitive value measure, respectively

**Fig. 5:**
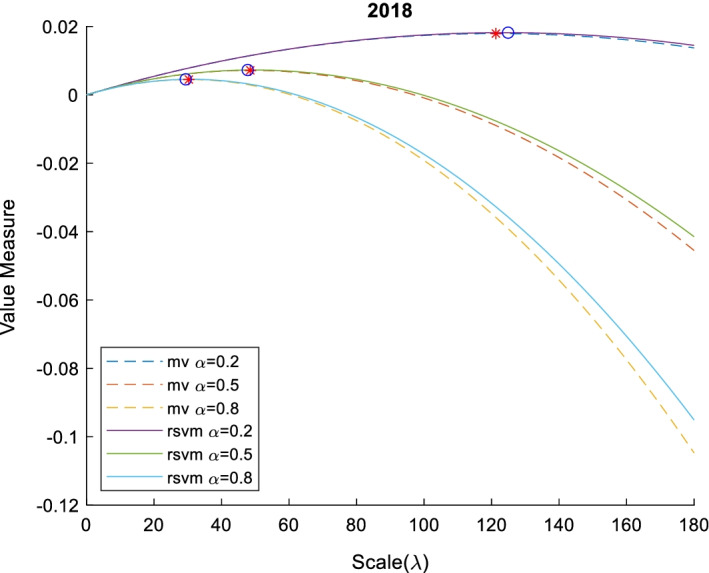
Value measure of RSVM and MV with various risk aversion $\alpha$ and scale $\lambda$ in 2018. This figure shows the relation between value measure (mean–variance model with dash line and risk-sensitive value measure with line) and scale with different investors (risk-averter $\alpha =0.8$, risk-neutral $\alpha =0.5$, and risk-seeker $\alpha =0.2$) in 2018. The red star and blue circle are optimal scale of mean–variance model and risk-sensitive value measure, respectively

**Fig. 6:**
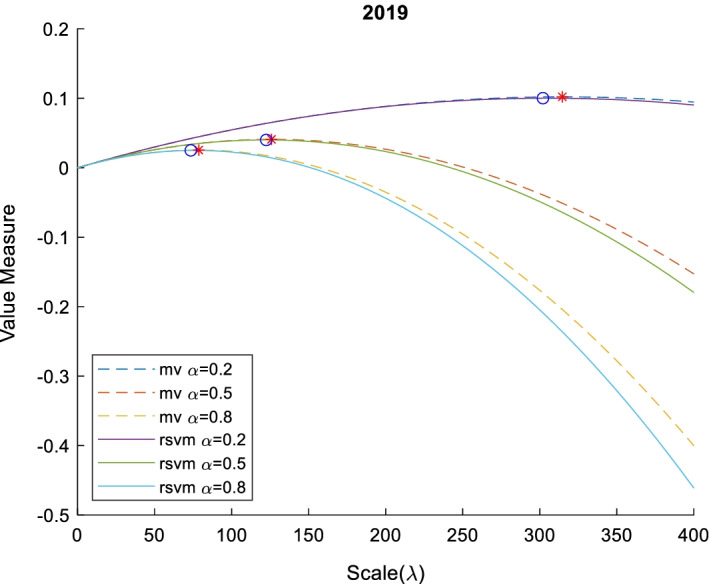
Value measure of RSVM and MV with various risk aversion $\alpha$ and scale $\lambda$ in 2019. This figure shows the relation between value measure (mean–variance model with dash line and risk-sensitive value measure with line) and scale with different investors (risk-averter $\alpha =0.8$, risk-neutral $\alpha =0.5$, and risk-seeker $\alpha =0.2$) in 2019. The red star and blue circle are optimal scale of mean–variance model and risk-sensitive value measure, respectively

**Fig. 7:**
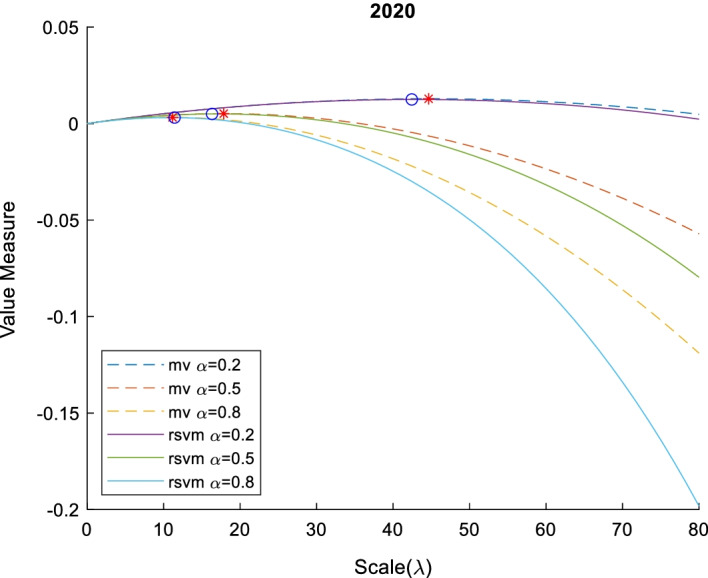
Value measure of RSVM and MV with various risk aversion $\alpha$ and scale $\lambda$ In 2020. This figure shows the relation between value measure (mean–variance model with dash line and risk-sensitive value measure with line) and scale with different investors (risk-averter $\alpha =0.8$, risk-neutral $\alpha =0.5$, and risk-seeker $\alpha =0.2$) in 2020. The red star and blue circle are optimal scale of mean–variance model and risk-sensitive value measure, respectively

As mentioned above, we can obtain a stressed portfolio using the semiparametric method with the optimal weights by minimizing the VaR of the portfolio. To further understand the scaling effect of the portfolio, we compare the mean–variance model and risk-sensitive value measure with different risk aversion, denoted by $\alpha$ from zero to one. We assume that there are three types of investors: risk-averter ($0.5<\alpha <1$), risk-seeker ($0<\alpha <0.5$), and risk-neutral ($\alpha =0.5$). We discuss the optimal scale of the portfolio during the five years with three types of investors, and the results are shown in Fig. 3, 4, 5, 6 and 7.

Although the curve of mean–variance (MV) and risk-sensitive value measure (RSVM) are similar in shape to a downward parabola, the curve of MV has a particularly strong concavity. In theory, the MV is a special case of an RSVM. MV has a close-form optimal portfolio scale shown in Eq. (4.3), while the optimal scale of the risk-sensitive value measure must be calculated by the Monte Carlo estimator. The numerical comparisons are listed in Tables 8 and 9.

**Table 8:** The optimal scale of portfolio with mean–variance model

Year	α = 0.2		α = 0.5		α = 0.8	
	Optimal scale	Value measure	Optimal scale	Value measure	Optimal scale	Value measure
2016	78.81104	0.009928	31.52442	0.003971	19.70276	0.002482
2017	344.2936	0.075148	137.7174	0.030059	86.0734	0.018787
2018	121.2824	0.017928	48.51295	0.007171	30.3206	0.004482
2019	314.6244	0.102044	125.8498	0.040817	78.65610	0.025511
2020	44.64709	0.012850	17.85884	0.005140	11.16177	0.003212

**Table 9:** The optimal scale of portfolio with risk-sensitive value measure

Year	α = 0.2		α = 0.5		α = 0.8	
	Optimal scale	Value measure	Optimal scale	Value measure	Optimal scale	Value measure
2016	79.59184	0.009933	30.61224	0.003971	20.40816	0.002480
2017	342.8571	0.075051	138.7755	0.030015	81.63265	0.018721
2018	124.8980	0.018188	47.75510	0.007267	29.38776	0.004536
2019	302.0408	0.100154	122.4490	0.040067	73.46939	0.025005
2020	42.44898	0.012502	16.32653	0.004991	11.42857	0.003110

The empirical results show a negative correlation between the degree of risk aversion and the optimal scale in the value measure. Risk-seeking investors correspond to larger scales, while risk-averters correspond to smaller scales. In addition, there is no difference in the mean–variance model and risk-sensitive value measure only for portfolios with a Gaussian distribution, but most portfolios are non-Gaussian in practice. If investors use a mean–variance model to determine the optimal scale, which may not be a real optimal scale, because the mean–variance model is not fit in a non-Gaussian distribution. Thus, the risk-sensitive value measure is pivotal in the stressed portfolio optimization.

## Conclusion

We propose an innovative semiparametric method for financial modeling and discuss the applications of portfolio optimization under tail risk with the scaling effect. This semiparametric method is composed of a nonparametric method and a copula method by estimating marginal distributions and the dependence of assets in a portfolio, respectively. Stressed portfolios and their optimal scaling effects are designed to be obtained by minimizing risk measures and maximizing risk-sensitive value measures, respectively. Through intensive empirical data analysis, we observe that the mean–variance type Markowitz method may cause bias selection, compared to the semiparametric method, which improves the efficiency of risk management with less risk exposure.

## Data Availability

Fortunately, our data is public because it comes from a data base company called Bloomberg.

## Abbreviations

MV: Mean–variance
VaR: Value-at-risk
CVaR: Conditional value-at-risk
RSVM: Risk-sensitive value measure
